# The effect of active virtual reality gaming on physical activity behaviour and mental health in young men with mild to moderate depressive symptoms: a randomised controlled feasibility trial

**DOI:** 10.1186/s12888-026-07904-6

**Published:** 2026-02-21

**Authors:** Fiona Hargraves, Joseph Firth, Emma George, Freya MacMillan, Sandra Garrido, Kerry A. Sherman, Allie Eathorne, Mike Armour

**Affiliations:** 1 NICM Health Research Institute, Western Sydney University, Westmead, NSW Australia; 2 Faculty of Medicine, Health and Human Sciences, Macquarie University, Sydney, NSW Australia; 3 Division of Psychology and Mental Health, University of Manchester, Manchester Academic Health Science Centre, Manchester, UK; 4 Greater Manchester Mental Health NHS Foundation Trust, Manchester Academic Health Science Centre, Manchester, UK; 5 Translational Health Research Institute, School of Medicine, Western Sydney University, Campbelltown, NSW Australia; 6 School of Health Sciences, Western Sydney University, Campbelltown, NSW Australia; 7 MARCS Institute for Brain, Behaviour & Development, Western Sydney University, Sydney, NSW Australia; 8 Lifespan Health and Wellbeing Research Centre, Macquarie University, Sydney, Australia; 9 School of Psychological Sciences, Macquarie University, Sydney, Australia; 10 Medical Research Institute of New Zealand, NZ, Wellington, New Zealand

**Keywords:** Mental health, Depression, PA, Virtual reality gaming, Digital technology, Exercise adherence, Health behaviour, COVID-19 trial, Online intervention

## Abstract

**Background:**

Physical activity (PA) benefits mental health, yet uptake and adherence are challenging, particularly for those affected by depression. Active virtual reality gaming (AVRG) may provide an engaging route to increase PA.

**Objective:**

To investigate the feasibility and acceptability of AVRG for increasing PA engagement and adherence in young men, and to explore effects on mental-health–related outcomes.

**Methods:**

In a randomised controlled feasibility trial (*n* = 30), physically inactive males aged 18–29, reporting mild to moderate depressive symptoms were allocated to Active AVRG (*n* = 14) or Waitlist (WL) control (*n* = 16). The intervention ran for 8 weeks with a 4-week post-trial follow-up. Exploratory analysis of secondary outcomes compared pre- and post-AVRG scores using paired t-test (normal), or Wilcoxon Signed-Rank test (non-normal); correlations used Pearson’s or Spearman’s coefficients.

**Results:**

Both the feasibility and acceptability criteria were met with 67% of potentially eligible participants, and 100% of those who booked phone screening randomised and enrolled. Retention was high, with 93.3% completing the study, and 87.5% completing all data collection measures. Exploratory analysis of secondary outcomes, showed a significant reduction in both Patient Health Questionnaire- 9 (PHQ-9) scores mean difference (MD) −2.82, (*p* = 0.006) and Depression, Anxiety and Stress Scale- 21 (DASS-21) stress scores MD −3.56 (*p* = 0.049) over the intervention period, as well as a very strong (*p* = 0.001) negative correlation (*r* =–0.57) between PHQ-9 score and number of sessions, a strong (*p* = 0.01) negative correlation between PHQ-9 and number of sessions > 30 mins. In addition, a moderate (*p* = 0.03) negative correlation (*r* =–0.43) was found between post-intervention DASS-21 Depression score and number of sessions > 30 mins. PA increased over the course of the intervention with none of the participants meeting the Australian National PA Guidelines at baseline, increasing to 50% at end of trial.

**Conclusions:**

This study found that a home-based AVRG intervention was feasible, acceptable, and safe for young men with mild–moderate depressive symptoms, with high recruitment, retention, and adherence. Exploratory findings indicate improvements in PA and favourable changes in mental-health measures. These results support progression to a fully powered trial.

**Trial registration:**

This trial was registered with the Australia and New Zealand Clinical Trials Register (approved 22/12/2020, Registration number: ACTRN12620001372976).

**Supplementary information:**

The online version contains supplementary material available at 10.1186/s12888-026-07904-6.

## Background

Depressive disorders are estimated to affect more than 350 million people globally [1] and in Australia around 7.5% of Australians reported an affective disorder [2] with the greatest increase in incidences of depression occurring before age 25, in adolescence and young adulthood.

Suicide is the leading cause of death for young Australians, accounting for almost one-third of deaths in those aged 15–24 years [2]. A compounding factor is that young people are also much less likely than any other age group to seek help, with 87% of young men with mental illnesses reporting not seeking medical or psychological help [3]. Evidence from during the COVID-19 pandemic showed depression and anxiety levels increased further during this period in young people; however, help-seeking behaviour did not [4]. As such, prevention in young adulthood currently presents a critical period, and indeed an opportunity, for early intervention.

The two depression treatments most recommended by medical practitioners are antidepressant medication and psychotherapy (cognitive behavioural therapy) [5, 6] and these both have noteworthy shortcomings, including the barrier of stigma to commencing treatment. As well as the low rates of help-seeking behaviour for mental health in young men [3] one of the biggest barriers to the success of depression interventions in this population is low acceptability and inconsistent adherence to the treatment [7].

The evidence for PA (PA) in reducing the risk of developing depression, protecting against future depressive episodes [8, 9] and in demonstrating antidepressant outcomes [10] is well established. This supports PA as a clear choice for clinicians in addressing depressive symptoms in patients and, in particular, for selecting modes of PA with high acceptability and accessibility. Exercise engagement and adherence to recommendations is the crucial factor in achieving and maintaining a reduction in depressive symptoms and preventing both future depressive episodes and cardiometabolic disease [11].

Internet based gaming is increasingly popular in the general population [12] and has evolved to be highly engaging and immersive with the advancement of augmented reality (AR) and virtual reality (VR) gaming. Active VR games, in which the user is physically active in their real environment as well as the virtual world allow users to play traditional sports such as tennis or golf, engage in fitness or dance sessions, in physical combat activities or games of skill designed especially for the VR environment. As VR gaming presents an accessible, scalable and acceptable opportunity to increase PA and enhance mental health, further research could inform the recommendations around VR as a PA and mental health management strategy, particularly for hard-to-reach populations.

Surveys conducted to inform the design of this trial focused on PA levels in young adults rather than mental health, yet the incidence of self-reported depressive symptoms was strikingly high. At the same time, COVID-19 lockdowns and facility closures restricted in-person activity options, but young men surveyed showed little interest in digital fitness formats such as synchronous or asynchronous online sessions which echoes other findings from this period [13].

The target group for this trial therefore sits between general mental wellbeing and diagnosed clinical depression, with a focus on young men who often do not report symptoms or seek formal help. The aim was to explore whether they could be reached and supported through a pragmatic intervention.

This study primarily aimed to examine the feasibility and effectiveness of AVRG on outcomes in PA engagement and adherence and depressive symptoms in young men affected by mild to moderate depressive symptoms. It was also designed to assess the influence on lifestyle factors, health behaviours and intentions, such as total PA engagement, sleep quality, exercise motivation and quality of life.

The primary objective of feasibility was evaluated via recruitment rate, engagement and adherence, retention, safety, and appropriateness of the outcome measures. A separate post-trial qualitative study was conducted to examine the facilitators and barriers experienced during this trial and this will be published separately.

The secondary objectives examined were measured via outcomes in depressive symptoms, health-related quality of life, exercise motivation, readiness to change PA behaviour, PA engagement, and sleep quality. Appendix A contains details of the outcome measures and assessment tools used.

## Methods

The trial consisted of an eight-week intervention where participants engaged in AVRG under pragmatic conditions; in their own homes at times and for durations they chose to engage. Participants were randomised into an Active and WL control group, who also engaged in the eight-week intervention after an eight-week waiting period. Data were collected between February 2021 and March 2022.

## Recruitment

Recruitment via Instagram advertisements led potential participants to an online self-screening eligibility survey. Only potentially eligible participants from the online self-screening were invited to provide contact details to progress to eligibility confirmation over the phone with the research team. Individuals with non-eligible or incomplete screening surveys were not contacted. Inclusion criteria were: being male; aged 18–29 years; physically inactive (a self-reported limit of up to 30 mins per week of moderate or greater intensity PA) [14]; had not been using an AVRG device within the last three months; were not undergoing any treatments to improve mood; and, had no medical contraindications to using the VR gaming device such as photosensitive epilepsy or implanted medical devices affected by magnets or radio waves.

All recruitment was undertaken in Australia with participants initially recruited via Instagram using hashtag-targeted posts (February 2021), followed by short paid “boosted” campaigns from July to early October 2021 until the target sample was reached. All advertisements directed interested individuals to the institute webpage.

Participants also needed to score between 5 and 14 on the PHQ-9 survey, indicating mild to moderate depression [15], and answer in the negative to the suicidal ideation question. In addition, they needed to complete the PA Readiness Questionnaire (PAR-Q) [16] to determine participant safety to engage in PA.

To minimise the potential for under or over-reporting PHQ-9 scores to be able to enter the trial, due to the desirability of AVRG, there was no information on eligible score, and no indication was provided to participants as to why they failed the screening.

The aim was a sample size of 30 based on evidence from similar feasibility and pilot trials, which showed median participant numbers are 30–36 and do not require formal sample size calculation [17, 18]. A sample size of 30 would also allow the intervention to be applied to both active and control groups in the randomised controlled trial (RCT).

### Consent and Ethics

Each participant was fully informed of the nature and objectives of the study and possible risks associated with participation. Then, written informed consent was obtained via an electronic form from each participant before any study-specific activity was performed (Appendices B and C).

Protecting participant personal data was ensured, and participant names were not included on any sponsor forms, reports, publications or in any other disclosures except where required by law. The informed consent form followed the International Conference on Harmonisation Good Clinical Practice Guidelines, local regulatory requirements and legal requirements. The Western Sydney University Human Ethics Committee granted approval for this study and use of this informed consent form on 27 November 2020 (H14118; Appendix C).

## Intervention

After being enrolled vis phone appointment by the research trial officer and completing baseline assessments, 30 participants were randomised in a 1:1 ratio to one of two groups: either Active or WL via REDCap. This system uses a computer-generated random number table with randomly permutated blocks with allocation sequence concealment. These features within REDCap for both sequence generation and allocation concealment reduce recruitment bias as neither the participants nor researchers know to which group a participant will be randomised [19]. After randomisation, the Active group were sent the trial kit containing the VR gaming device (Oculus Quest 2), a Polar heart rate monitor (chest strap model), and instructions for setting up the equipment (hardware) and downloading the software (games). Those randomised to the WL group received this after their eight-week waiting period. Details of the technology used are contained in Appendix E.

During an eight-week intervention period, participants were given access to three commercially popular active VR games, Thrill of the Fight, Pistol Whip and Beat Sabre. These games were chosen due to their PA equivalent ratings of tennis or higher, as per the Virtual Reality Institute of Health and Exercise [20]. Participants were recommended to engage in a minimum dose of 90 minutes per week (three 30-minute sessions in accordance with the VR equipment manufacturer’s guidelines for maximum gaming session duration) of AVRG, with no upper limit set on number of sessions per week. This aligns with the *Australian PA Guidelines*’ 75–150 minutes of vigorous-intensity activity [21]. To track gaming engagement, participants were instructed on setting up tracking on the Oculus Quest gaming system and also using the provided heart rate monitor, connected via Bluetooth to VR Health Exercise Tracker app - created by the Virtual Reality Institute of Health and Exercise - on their personal smartphone.

The Oculus Quest 2 was selected after assessing comparable devices primarily for safety features: a wireless headset and a ‘guardian’ system with external cameras that alerts users when approaching the two metre by two metre physical play space (Appendix E). In keeping with the pragmatic design, no constraints were placed on engagement beyond PA dose recommendations. As the games do not include warm-ups or cool-downs, adverse events were monitored. Although games are single player by design, participants could play alongside others in the room or via online modes to reflect real-world use.

## Outcome assessments and data capture

The trial was run fully online, and data were captured electronically via the secure electronic clinical research database platform REDCap [22]. The total study duration of the intervention phase was 13 weeks: 1 week for baseline measures and equipment delivery; eight weeks of active treatment; and four weeks of follow-up. For the WL control group, the total study duration was 22 weeks including the eight-week waiting period.

At the completion of the intervention period, all participants were requested to upload screenshots of gaming data tracked by the VR Exercise app and the Oculus Quest Move app to REDCap for engagement and adherence measures.

### Primary outcome measures—feasibility

## Recruitment rate

The recruitment rate was calculated as the percentage of individuals who were successfully enrolled out of those who were screened as potentially eligible from the screening questionnaire (the number of participants who were randomised from those who were eligible to join) and, second, the percentage of individuals who were successfully enrolled in the trial out of all who expressed interest in the study by completing the screening questionnaire (the number of participants who were randomised from all those who were interested in joining the study). These outcomes report interest in participating in the trial, appropriateness of recruitment strategies and the eligibility criteria.

## Engagement and adherence

Compliance was defined as the proportion of participants who achieved three 30-minute sessions per week and the number of weeks for which this threshold was met, to indicate both initiation and sustained participation in PA. There was potential for human error in session data captured with Oculus Move and the VR Exercise app. As such, the data collected were analysed using the following guidelines:

- If only one source recorded a session, that value was used.
- If both sources recorded the session with the same value, that value was used; if values differed, the VR Exercise minutes took precedence because Oculus Move reports progress with a pie-chart indicator rather than minutes.
- When only Oculus Move data were available, it was assumed the Move goal had been configured as instructed: a complete ring indicated at least thirty minutes and an incomplete ring indicated fewer than thirty minutes, unless there was evidence that a different goal had been set (participant report or device inspection).
- If neither source showed a session, none was logged.
- Sessions were assigned to the calendar day and week performed.
- Sessions lasting at least thirty minutes counted toward the weekly recommendation; sessions shorter than thirty minutes were recorded as engagement but did not contribute to compliance.

## Retention

Dropout rates were calculated as the percentage of participants to complete both the baseline and endpoint outcome measures.

## Safety

During both WL and active intervention periods, participants received a weekly REDCap email survey asking about any health changes in the previous seven days. The survey monitored external conditions that could affect gaming (including COVID-19) and any adverse events related to the intervention. Reports were graded by the trial medical monitor using the National Cancer Institute Common Terminology Criteria for Adverse Events version 6.0 [23]. Although AVRG is generally low risk, participants were inactive at enrolment and undertook moderate to vigorous activity; monitoring ensured any undesirable effects were detected.

The midpoint health survey for both periods also included the PHQ-9 depressive-symptom measure to support safety monitoring and identify any participants with deteriorating mental health or a positive response to the suicidal ideation item (an exclusion criterion) to follow up if required.

## Appropriateness of the outcome measures—compliance with data collection

Participants were instructed to configure the Oculus Move app on the Quest 2 and to activate the VR Exercise smartphone app and heart-rate monitor, to be used with every AVRG session. Data-collection compliance was defined as completion of the weekly health-monitoring survey and upload of session screenshots to REDCap from Oculus Move, VR Exercise, or both. The surveys also recorded illnesses or injuries unrelated to the intervention (for example, COVID-19) that could affect participation.

### Secondary outcome measures

## Depressive symptoms

The Patient Health Questionnaire-9 (PHQ-9;15) was used to screen and quantify depressive symptoms because it is brief, valid for online administration, and sensitive to change, with strong psychometric support [24]. The Depression Anxiety Stress Scales-21 (DASS-21;[25]) assessed symptoms of depression, anxiety and stress on separate subscales; it is suitable for clinical and non-clinical settings and demonstrates high internal consistency and structural validity [26] and is recognised as being reliable and easy to administer [27].

The PHQ-9 was completed at baseline prior to randomisation, mid-intervention (week four), and endpoint (week nine) for all participants; WL participants also completed PHQ-9 at week four of the waiting period and again at baseline two (pre-intervention). DASS-21 was completed at baseline and endpoint for all participants and again by the WL group at baseline two.

Scoring and missing data: For the PHQ-9, if more than one item was missing the scale score was set to missing; up to one missing response was permitted [28]. PHQ-9 scores were interpreted according to standard guidelines: ≤ 4 = minimal depression; 5–14 = mild to moderate depression; > 14 = moderately severe to severe depression.

For the DASS-21, one missing item per seven-item subscale was permitted; otherwise the subscale score was not calculated [29]. DASS-21 scores were interpreted according to standard severity ranges: Normal (Depression = 0–4; Anxiety = 0–3; Stress = 0–7); Mild (Depression = 5–6; Anxiety = 4–5; Stress = 8–9); Moderate (Depression = 7–10; Anxiety = 6–7; Stress = 10–12); Severe (Depression = 11–13; Anxiety = 8–9; Stress = 13–16); Extremely Severe (Depression ≥ 14; Anxiety ≥ 10; Stress ≥ 17).

## Physical activity engagement

The International PA Questionnaire (IPAQ; 30) is a brief, self-administered seven-day recall of incidental and planned PA. Activity trackers were not used due to budget and logistics constraints; IPAQ was considered appropriate given baseline recruitment for physical inactivity and separate tracking of gaming sessions. The IPAQ is widely used in intervention research, and online administration in young people during COVID-19 has shown acceptable reliability [30]. IPAQ was completed at baseline before randomisation, at endpoint after the AVRG intervention for all participants, and again by the WL group at baseline two. For scoring IPAQ, cases with missing days or minutes, or “don’t know” entries, were removed from analysis. Then, activity minutes were converted to MET (Metabolic Equivalent Task)-minutes per week (METs are multiples of resting metabolic rate) by categorising them by intensity and multiplying the minutes by the corresponding MET value: low/walking (3.3 METs), moderate (4 METs), or high/vigorous (8 METs). Categorical scores were determined according to three levels [31]:

Category 1, Low: This is the lowest level of physical activity. Those individuals who do not meet criteria for categories 2 or 3 are considered low/inactive.

Category 2, Moderate: Any one of the following three criteria;

- 3 or more days of vigorous activity of at least 20 minutes per day.
- 5 or more days of moderate-intensity activity or walking of at least 30 minutes per day.
- 5 or more days of any combination of walking, moderate-intensity or vigorous intensity activities achieving a minimum of at least 600 MET-min/week.

Category 3, High: Any one of the following two criteria;

- Vigorous-intensity activity on at least 3 days and accumulating at least 1500 MET-minutes/week.
- 7 or more days of any combination of walking, moderate-intensity or vigorous intensity activities achieving a minimum of at least 3000 MET-minutes/week.

## Physical activity motivation

The Exercise Motivation Inventory-2 (EMI-2; [32]) assesses motives for exercise and non-participation across a broad range of domains and is appropriate for adult exercisers and non-exercisers, with evidence supporting reliability [33]. This tool was used to detect changes in thoughts, feelings and motivations about PA during the intervention. The EMI-2 was completed at baseline prior to randomisation and endpoint after the AVRG intervention for all participants and again by the WL group at baseline two.

Participants rated statements on fourteen subscales from zero, ‘not at all true for me’, to five, ‘very true for me’. For scoring, if any item was missing for a subscale, no score was calculated for that subscale. Higher scores indicate higher motivation.

## Readiness to change

A single question in the Stage of Change (SOC) format (Appendix A) assessed readiness for PA change using the Transtheoretical Model (TTM) [34]. At baseline, most participants were expected to be in precontemplation or contemplation (selections one and two on the question below); movement to action or maintenance would indicate improved engagement (selections three-five on the question below). This assessment was completed at baseline before randomisation and at endpoint after the AVRG intervention for all participants, and again by the WL group at baseline two. Missing responses were treated as missing data.

## Quality of life

The Short Form-36 (SF-36; [35]) is a well-validated assessment of mental and physical health with eight scales: physical functioning, physical role functioning, social functioning, mental health, emotional role functioning, vitality, bodily pain and general health. It has shown excellent internal consistency and construct validity, providing a reliable indication of health-related quality of life [36, 37]. This instrument was used to examine changes in specific aspects of functioning during the intervention. Participants completed the SF-36 at baseline before randomisation and at endpoint after the AVRG intervention, and the WL group completed the measure again at baseline two. For scoring the SF-36, responses are transformed to a 0–100 rating for eight different health scales, where a higher score indicates better subjective mental and physical wellbeing [38].

## Sleep

The Pittsburgh Sleep Quality Index (PSQI; [39]) is a self-rated measure of sleep quality and disturbance over one month, comprising seven components: subjective sleep quality, sleep latency, sleep duration, habitual sleep efficiency, sleep disturbances, use of sleeping medication, and daytime dysfunction. Studies examining the psychometric properties of the PSQI have found it a reliable and valid scale for assessing sleep quality [40]. The PSQI was included to characterise lifestyle and because PA can improve sleep [41]. Participants completed this assessment at baseline before randomisation and at endpoint after the AVRG intervention, with the WL group completing it again at baseline two. Component scores were set to missing if any required item was missing. A global score was calculated, with lower values indicating better sleep quality. For scoring the PSQI, the seven component scores, each ranging from zero to three, are added to get a global score from zero to 21. Each component and the global score are calculated by assigning a score of zero for no difficulty to three for severe difficulty. Higher global scores indicate poorer sleep quality, with a score greater than five suggesting significant sleep difficulties [39].

## Demographics and lifestyle

Demographic information collected at baseline included age, gender, education status, employment status, postcode of residence and how many people reside there. Lifestyle and health behaviour data collected included time spent on active and non-active gaming, platforms used and perceived barriers to, importance of and confidence in PA participation.

## Trial schedule

See Table 1 for outcome measures by timepoint and Appendix A for further trial outcome details. A flow chart of trial activities is included in Appendix F.

**Table 1:** Outcome measures by timepoint

Recruitment period
Recruitment rate
**Intervention baseline and endpoint**
Retention
Depressive symptoms
PA engagement
Exercise motivation
Readiness to change
Quality of life
Sleep quality
**Intervention endpoint**
Engagement and adherence
Participant satisfaction
**Intervention & WL baseline and endpoint and four-week follow-up.**
Participant lifestyle
**Intervention baseline, midpoint and endpoint and WL baseline and endpoint.**
Depressive symptoms
**Weekly during WL and intervention periods**
Safety – adverse events
**Weekly during WL and intervention periods and post-intervention**
Compliance with data collection

See Tables 2 and 3 for the trial schedule.

**Table 2:** Schedule of events—active group

Period	S		Intervention								
Week	0	1, O, B	2	3	4	5 M	6	7	8	9 E	13, F
Eligibility screening	X										
Informed consent signed	X										
Randomisation		X									
Demographics		X									
Feasibility measures										X	
PHQ-9		X				X				X	
DASS-21		X								X	
EMI-2		X								X	
Readiness to change		X								X	
PSQI		X								X	
SF-36		X								X	
Adverse events		X	X	X	X	X	X	X	X	X	
Participant satisfaction										X	
Follow-up survey											X

Note: S = Screening O = Orientation (equipment set-up), B = Baseline, F = Follow-up, *M* = Midpoint, E = Endpoint

**Table 3:** Schedule of events—WL group

Period	S		WL		O	Intervention												
Week	0	B1	W 2–8	E, B2, 9	10	11	12		13 M		14		15	16	17	18 E		F 22
Eligibility screening	X																	
Informed consent signed		X																
Randomisation		X																
Demographics		X																
Feasibility measures																X		
PHQ-9		X		X				X								X		
DASS-21		X		X												X		
EMI-2		X		X												X		
Readiness to change		X		X												X		
PSQI		X		X												X		
SF-36		X		X												X		
Adverse events				X	X	X	X	X		X		X		X	X	X	X	
Participant satisfaction																X		
Follow-up survey																	X	

Note: S = Screening, O = Orientation (equipment set up), B = Baseline, W = Week, *M* = Midpoint, E = Endpoint, F = Follow-up

## Statistical analysis

Baseline differences between groups were examined using independent t-tests for continuous variables with approximately normal distributions (e.g., age), Mann–Whitney U tests for skewed or ordinal variables (e.g., household size), and chi-square or Fisher’s exact tests for categorical variables, as appropriate.

Differences in pre- and post-AVRG scores were estimated by paired t-test, or where normality assumptions not well met (checked by residual distributions and plots of residuals versus predicted values) Wilcoxon Signed-Rank test. Analyses were performed using Statistical Analysis System (SAS) software version 9.4, Copyright © 2016 SAS Institute Inc., Cary, NC, USA.

The study was designed to examine only within-group changes as it is a feasibility study (not efficacy) rather than between-group changes due to the lack of power.

An exploratory correlation analysis undertaken to identify any relationships existing between the depressive symptom outcomes was performed using Pearson’s rank unless marked as Spearman’s rank (see Appendix G).

The four-week follow-up contained questions on participant lifestyle and continued or intended engagement in AVRG only as participants did not have access to trial equipment after the conclusion of their intervention period.

## Results

### Participant demographics

The trial participants (*n* = 30) were all men located in urban areas and had a median age of 23 years (range = 18–29). Groups did not differ significantly in age (*p* = 0.34). Household size was larger in the WL group compared with the active group (*p* = 0.015). Around one-third of participants were undertaking tertiary education (one participant was enrolled in high school, not reflected in the categories of Table 6), with enrolments spread across on-campus, online, part-time, and full-time modes. Approximately two-thirds of participants were employed at least part-time, with no significant differences between groups in employment status, education, or study load (See Table 4).

**Table 4:** Participant Demographics

**Characteristic** (**baseline)**	Total sample, n = 30	Active, n = 14	WL, n = 16	p-value
**Age**				0.34
Mean (SD)	23.4 (3.7)	24.1 (3.7)	22.8 (3.5)	
Range	18–29	18–29	18–28	
**Total no. people at place of residence**				0.015
Mean (SD)	3.2 (1.5)	2.4 (1.2)	3.9 (1.5)	
Range	1–6	1–4	1–6	
**Location** (n, %)				NA
Urban	30 (100)	14 (100)	16 (100)	
Rural, remote	0 (0)	0 (0)	0 (0)	
**Employment Status** (n, %)				0.98
Working full-time (>35 hours per week)	12 (40)	5 (35.5)	7 (44)	
Working part-time (<35 hours per week)	10 (33.3)	5 (35.5)	5 (31)	
Self-employed, volunteer/unpaid	0 (0)	0 (0)	0 (0)	
Not working	8 (26.7)	4 (28.5)	4 (25)	
**Tertiary Study**				0.56
None	18 (60)	9 (64)	9 (56.3)	
Diploma	1 (3.33)	0 (0)	1 (6.3)	
Bachelor’s degree	9 (30)	4 (29)	5 (31)	
Graduate certificate	1 (3.33)	1(7)	0 (0)	
Doctoral degree	1 (3.33)	0 (0)	1 (6.3)	
Graduate certificate, advanced diploma, associate degree, bachelor honours degree, graduate diploma, master’s degree	0 (0)	0 (0)	0 (0)	
**Study Mode (of those studying, n = 12)**	(*n* = 12)	(*n* = 5)	(*n* = 7)	0.29
Online/distance due to COVID-19	6 (50)	2 (40)	4 (57)	
Enrolled in online/distance course	3 (25)	2(40)	1 (14)	
Blended; on campus and online/distance	1 (8.3)	1 (10)	0 (0)	
On campus	2 (16.7)	0 (0)	2 (29)	
**Study Load (of those studying, n = 12)**	(*n* = 12)	(*n* = 5)	(*n* = 7)	1.00
Full-time	9 (75)	4 (90)	5 (71)	
Part-time	3 (25)	1 (10)	2 (29)	

Notes: SD = Standard Deviation. NA = Not Applicable

**Table 5:** Engagement and adherence tracked during the eight-week intervention period

Number of sessions tracked > 30 minutes in length	Mean (SD)	95% CI
All participants (*n* = 28)	11.25 (9.4)	7.61–14.89
Active group (*n* = 14)	8.4 (8.9)	3.29–13.51
WL group (*n* = 14)	14.1 (9.4)	8.67–19.53
**Total number of sessions tracked of any length**	**Mean (SD)**	**95% CI**
All participants (*n* = 28)	15.4 (10.9)	11.18–19.62
Active group (*n* = 14)	11.9 (10.7)	5.72–18.08
WL group (*n* = 14)	18.9 (10.2)	13.01–24.79

Notes: CI = Confidence Interval

**Table 6:** AVRG trial outcome measure compliance

Compliance activity	Active n = 14 n (%)	WL (n = 16) n (%)
No missing data, upload from **both** tracking apps, all surveys	7 (50)	8 (50)
Weekly surveys completed all	10 (71)	11 (68.8)
Midpoint PHQ-9 complete	13 (92.9)	16 (100) ^a/^ 14 (93.3) ^b^
**Uploaded screenshots**		
Both apps	9 (64.3)	9 (56.3)
Oculus Move app	11 (78.6)	11 (68.8)
VR Exercise app	11 (78.6)	12 (75)
From either app	13 (92.9)	14 (87.5)
From neither app	1 (7)	2 (12.5)

^a^Midpoint PHQ-9 during WL period

^b^Midpoint PHQ-9 during active period (*n* = 15) due to one withdrawal at that point

## Primary outcomes

### Recruitment rate

**Fig. 1:**
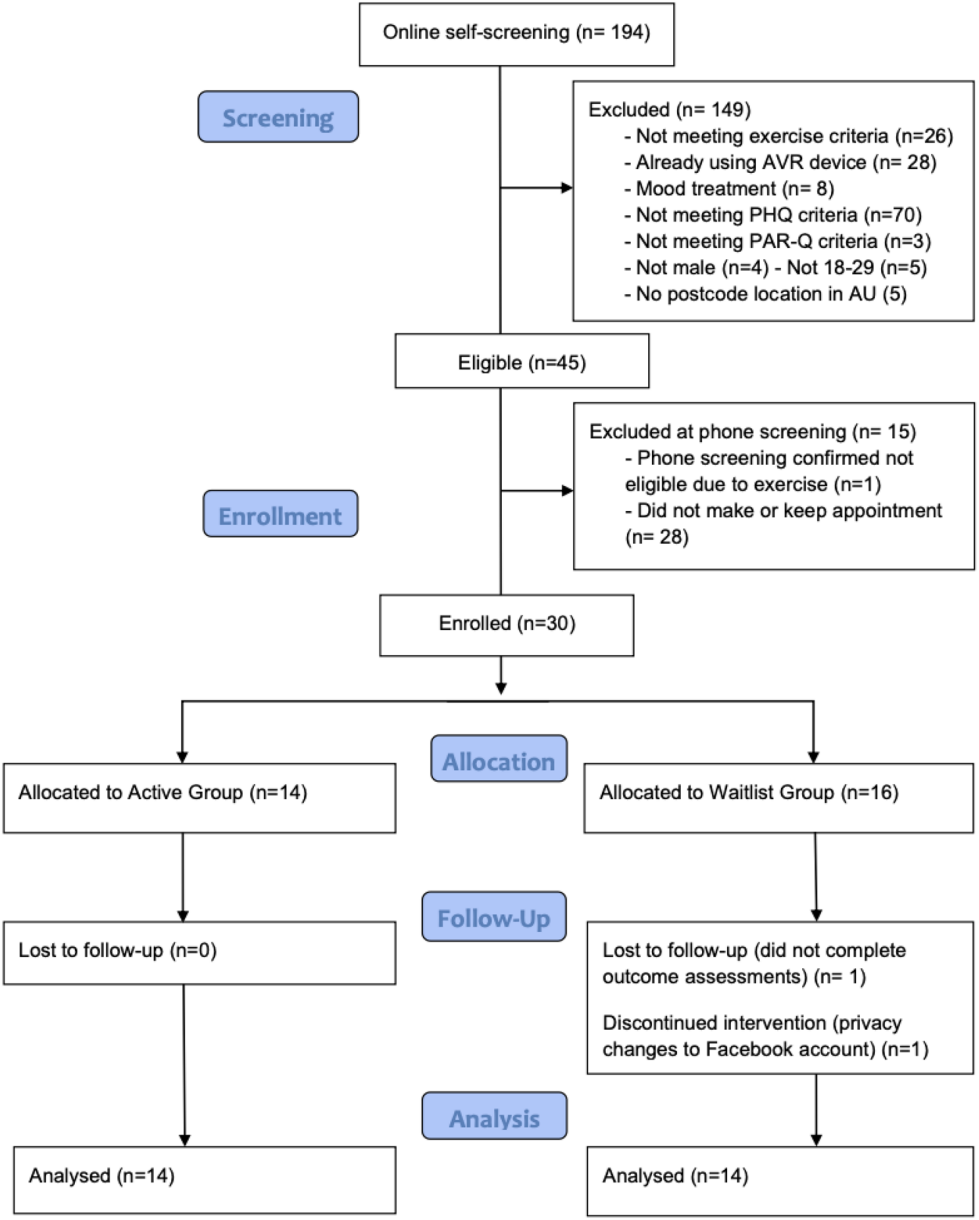
Recruitment

Recruitment and enrolment from February 2021 to October 2021 resulted in 194 completed screening surveys and 45 potentially eligible participants of which 31 (69%) booked in for and attended a phone appointment to confirm eligibility and enrol in the trial. Of those 31, 100% of those who had correctly completed the pre-screening were enrolled at the phone appointment. Recruitment was terminated after the 30th participant was enrolled and randomised. As such, the recruitment rate was 67% of participants randomised (*n* = 30) out of those potentially eligible and 15.5% (*n* = 194) out of those expressing interest via self-screening completion (see Fig. 1).

Participants completed their eight-week intervention between February 2021 and March 2022.

### Engagement and adherence

Due to discrepancies between data recorded by the Oculus Move app and VR Exercise app, it was evident participants did not use both tracking methods consistently. As such, engagement and adherence to the active VR gaming was determined as the number of sessions of at least 30 minutes ‘*tracked’* rather than the number of sessions undertaken, and the total engagement – which may have been higher - may not have been captured in many cases (Table 5). The results show the WL group tracked more gaming than the Active group.

To investigate potential differences in gaming adherence between groups, and because normality assumptions were not adequately met, adherence variables were analysed using a Mann–Whitney U test with the Hodges–Lehmann estimator for the location difference and associated 95% confidence intervals. No significant between-group differences were detected. For sessions > 30 minutes, the Hodges–Lehmann estimate for the Active minus Waitlist comparison was −7.0 (95% CI: −15.0 to 1.0; *p* = 0.10). For sessions of any length, the estimate was −8.0 (95% CI: −16.0 to 1.0; *p* = 0.07).

### Retention

Retention was high across the trial (93.3% overall). A total of 30 participants were randomised (Active = 14; Waitlist = 16), and 28 completed the study. All participants in the active group (100%) completed the intervention, while two participants in the WL group did not complete, yielding an 87.5% retention rate in that arm. One withdrew following the WL period, citing concerns about Facebook and Oculus Quest accounts becoming part of the Metaverse platform, and one was lost to follow-up. Importantly, no withdrawals were due to adverse experiences of the intervention itself. Attrition was therefore minimal and confined to the WL arm, suggesting high acceptability of the intervention and reducing the risk of attrition bias.

### Safety—adverse events

There were only two incidents assessed by medical monitors as possibly/probably related to the trial activities. One case of muscle soreness and one case of mild chest muscle strain, from a participant playing an intense game not provided as part of the trial.

Most participants in the WL group reported having COVID-19 at some stage during their active intervention, some reported during the trial, however some only reported this on follow-up.

### Appropriateness of the outcome measures—compliance with data collection

Overall compliance with data collection for this trial was defined as completing weekly health surveys and uploading screenshots of tracked game playing to the REDCap database at the completion of the intervention period (see Table 6).

### Participant satisfaction

In endpoint surveys, participants reported very high enjoyment in the gaming, and user-friendliness for the data collection technology, further indicating acceptability in this demographic (see Table 7).

**Table 7:** AVRG trial participant satisfaction

Satisfaction	n	Median (IQR)
How **enjoyable** did you find the trial activities of active VR gaming? (1 = not enjoyable, 10 = extremely enjoyable)	28	9 (7.5–10)
How likely would you be to **continue** AVRG if you had continued access to the Oculus Quest 2 device? (1 = not likely, 10 = extremely likely)	28	9 (6–10)
How likely would you be to **recommend** participation in AVRG to family and friends? (1 = not likely, 10 = extremely likely)	28	8 (7.5–10)
How **user-friendly** did you find the process of wearing a heart rate monitor connected to the **VR Exercise app** for each gaming session during this trial? (1 = not user-friendly, 10 = extremely user-friendly)	27	8 (6–10)
How **user-friendly** did you find the process of undertaking **outcome assessments** via the REDCap app during this trial? (1 = not user-friendly, 10 = extremely user-friendly)	27	8 (6–10)

## Secondary outcomes

For the active intervention period data analysis, pre-randomisation baseline assessments were used for the active group and baseline 2 data for the WL group as this was their pre-intervention baseline assessment. Both groups were sent the VR equipment to begin the gaming intervention directly after completing these respective baselines.

The trial was designed to examine only within-group changes as it was a feasibility study rather than between-group changes due to the lack of power. Appendix G contains secondary outcome data for the active and WL periods referred to in the summaries below, and data were analysed as detailed in the Statistical Analysis section above.

### Depressive symptoms and the relationship between PA and mental health

There was a significant reduction (MD −2.82, 95% CI − 4.77 to −0.87, *p* = 0.006) in PHQ-9 scores between baseline and end of trial after undertaking the AVRG intervention, with lower PHQ-9 scores indicating improvements in mood.

There was also a significant reduction (MD −3.56, 95% CI − 7.09 to −0.02, *p* = 0.049) in the DASS-21 Stress scores between baseline and end of the trial with lower scores indicating a reduction in stress; participants in this trial moved from the severe to mild stress category during the intervention [25].

All depressive symptom scores (PHQ-9 and DASS-21 sub-scales) decreased, even if not by a statistically significant margin, indicating participants in both groups moved in the favourable direction for testing the feasibility of AVRG on depressive symptoms (Table 8).

**Table 8:** Changes in depressive symptoms between baseline and end of the active phase of the intervention for all participants

Variable	N	Mean (SD)	95% CI	Median (IQR)	Min to Max
Pre-PHQ-9	28	8.8 (4)	7.32–10.28	8.5 (6 to 11.5)	1 to 19
Post-PHQ-9	28	6 (4.4)	4.37–7.63	5.5 (2 to 9)	1 to 20
Pre-DASS-21 Anxiety	28	7.1 (5.2)	5.17–9.03	6 (4 to 10)	0 to 20
Post-DASS-21 Anxiety	27	5.4 (5.5)	3.33–7.47	4 (0 to 6)	0 to 22
Pre-DASS-21 Depression	27	11.2 (6.4)	8.79–13.61	10 (6 to 16)	2 to 26
Post-DASS-21 Depression	26	7.4 (7.2)	4.63–10.17	7 (2 to 10)	0 to 28
Pre-DASS-21 Stress	27	13 (8.4)	9.83–16.17	12 (8 to 18)	0 to 30
Post-DASS-21 Stress	28	9.4 (7.7)	6.55–12.25	10 (3 to 12)	0 to 30

Notes: PHQ-9 scoring: ≤ 4 = minimal depression; 5–14 = mild to moderate depression; > 14 = moderately severe to severe depression. DASS-21 scoring; Normal (Depression = 0–4; Anxiety = 0–3; Stress = 0–7); Mild (Depression = 5–6; Anxiety = 4–5; Stress = 8–9); Moderate (Depression = 7–10; Anxiety = 6–7; Stress = 10–12); Severe (Depression = 11–13; Anxiety = 8–9; Stress = 13–16); Extremely Severe (Depression ≥ 14; Anxiety ≥ 10; Stress ≥ 17)

There was no significant difference in the PHQ-9 scores in the WL group between baseline and end of waiting period, as displayed in Appendix G (pre- minus post-WL scores). There were no significant differences in the WL group between baseline and end of the waiting period for DASS-21 Anxiety, DASS-21 Stress, or DASS-21 Depression, as displayed in Appendix B (pre- minus post-WL scores).

The exploratory correlation analysis (Table 9) demonstrated a statistically significant very strong negative correlation (*r*=–0.57, *p* = 0.001) between post-intervention (week 9) PHQ-9 score and total number of sessions, and a strong negative correlation between post-intervention PHQ-9 and number of sessions > 30 minutes (*r* = −0.47, *p* = 0.01). In addition, a moderate negative correlation (*r*=–0.43, *p* = 0.03) was found between post-intervention (week 9) DASS-21 Depression score and number of sessions > 30 minutes.

**Table 9:** Cross-correlation matrix of AVRG exposure metrics and mental-health outcomes

	Number of sessions > 30 mins	Total number of sessions	Week 9 PHQ-9	Baseline stage of change	Week 9 DASS Anxiety	Week 9 DASS Depression	Week 9 DASS Stress
Number of sessions > 30 mins	-						
Total number of sessions	0.86 **(<0.0001)**	-					
Week 9 PHQ-9	−0.47 **(0.01)**	−0.57 **(0.001)**	-				
Baseline stage of change	−0.07^s^ (0.72)	−0.05^s^ (0.80)	0.04^s^ (0.85)	-			
Week 9 DASS Anxiety	−0.19 (0.34)	−0.22 (0.28)	0.60 **(0.001)**	−0.09^s^ (0.65)	-		
Week 9 DASS Depression	−0.43 **(0.03)**	−0.36 (0.07)	0.74 **(<0.0001)**	0.09^s^ (0.67)	0.63 **(0.0008)**	-	
Week 9 DASS Stress	−0.29 (0.14)	−0.25 (0.20)	0.57 **(0.002)**	0.21^s^ (0.29)	0.55 **(0.003)**	0.74 **(<0.0001)**	-

Correlations are Pearson’s, except where denoted as Spearman’s by ^S^. *p*-values are in brackets, significant results (at 5% significance level) in bold.

The cross-correlation matrix (Table 9) summarises pairwise associations among exposure metrics and symptom measures at week nine. As noted above, depressive symptoms were inversely related to total sessions and to sessions lasting at least thirty minutes; other correlations were not statistically significant. Exact coefficients and *p*-values are provided in Appendix G (Pearson’s or Spearman’s, two-sided).

### PA engagement and motivation

There was a significant increase (median difference 0.05, 95% CI 0.00 to 1.00, *p* = 0.03) in IPAQ scores - incidental and planned PA - between baseline and end of trial, with higher scores indicating an increase in PA as shown in Appendix G.

There were no significant differences in exercise motivation on the 14 sub-scales of the EMI-2 pre- and post-intervention (Appendix G).

During the waiting period for the WL group, there was a moderately significant increase (MD 0.46, 95% CI 0.91 to 0.02, *p* = 0.04) in EMI-2 Challenge scores between baseline and end of the waiting period, indicating participants experienced an increase in psychological motives related to working towards goals. There were no other significant differences in outcome measures between baseline and end of the waiting period for the WL group.

### Readiness to change, quality of life and sleep

The simple one-question SOC format, evaluated readiness to change PA behaviour based on the stages in the TTM [34]. A higher score indicates a greater readiness to change PA behaviour (Appendix G). There was a significant increase (MD 0.57, 95% CI 0.16 to 1.00, *p* = 0.009) in SOC scores between baseline and end of the trial (Appendix G).

The SF-36 examined whether there were any changes in particular aspects of physical and mental health and functioning during the intervention [35] (Appendix G). There was a significant increase (MD −10.65, 95% CI 2.77 to 18.54, *p* = 0.01) in SF-36 energy/fatigue scores over time between baseline and end of trial. An increase in this score indicates improvement in subjective wellbeing and vitality [38]. This increase moved participants, as a group, from significantly below normative (55% of norm) to closer to normative values (70% of norm) for energy/fatigue (vitality) for Australian males aged 18–34 [42]. There was a significant increase in SF-36 role functioning/emotional scores between baseline and end of the trial (median difference 33.33, 95% CI 0.00 to 66.67, *p*=< 0.001). See Appendix G for all outcomes.

There was no significant difference in pre- and post-intervention scores on the PSQI; however, the decrease in mean scores indicates sleep quality moved in the direction of sleep improvement over the eight weeks (see Appendix G).

### Participant lifestyle

At baseline, participants reported substantially more non-active gaming than post-intervention; the proportion playing more than three hours per week fell from 57% to 29%. No participant met the Australian weekly physical-activity recommendation at baseline [21]. By endpoint, 50% reported at least moderate-intensity PA totalling 60–120 mins, 120–180 mins or > 180 mins per week, thereby meeting the guideline threshold (Appendix H).

The four-week post-intervention follow-up survey, regarding participants’ intended and ongoing gaming and lifestyle habits, was completed by 50% (*n* = 15) of participants. Of these, 20% reported continued AVRG and a further 53.3% said they would if they had access to a VR device, as the trial equipment was collected at the conclusion of the intervention period (Appendix H). This follow-up was designed to capture any changes in PA post-intervention - including any future interest in AVRG. As the participants had returned their AVRG equipment, this questionnaire was not designed to track any AVRG specific changes.

## Discussion

This trial demonstrated the feasibility of AVRG as a potentially highly acceptable and engaging PA intervention in a population of young males, a demographic typically difficult to engage in PA interventions [43]. This RCT achieved high levels of conversion from expression of interest to enrolment and randomisation, a very high retention rate and good compliance with data collection, highlighting the potential advantages of using a remote, decentralised approach in this population. The extremely high participant satisfaction scores add to the evidence of the intervention activity acceptability in the target population and validate the potential of using VR gaming to keep young men more engaged in PA in larger RCTs and in real life, especially those who are physically inactive with low mood. The acceptability shown in this trial validates the evidence for young men’s preference for modes of PA focused on mastery and enjoyment [44] as well as the attraction of new technology [45]. These feasibility results also emphasise the link between enjoyment and exercise habit, intention to continue and frequency demonstrated in studies investigating the intention–behaviour gap in exercise practice [46]. The recruitment success and feasibility outcomes observed in this study also provide valuable guidance for the design of future AVRG trials. High rates of interest, enrolment, retention, compliance and enjoyment suggest that remote, decentralised approaches can be highly effective in engaging young men in research settings that traditionally struggle to recruit this demographic. Future studies can build on these strategies by refining online recruitment methods, leveraging social media platforms, and minimising participant burden in data collection. Such approaches may enhance scalability, improve representativeness, and strengthen the evidence base on the role of AVRG in promoting PA and mental health.

This feasibility trial was not designed or powered to collect economic data. The study was delivered remotely by a single investigator, with REDCap configured by a contractor, and the primary focus was on recruitment, retention, adherence, safety and data completeness. Given the very small sample and atypical pandemic context—along with rapid price volatility for VR hardware and software—point estimates of cost would have been unstable and potentially misleading. We therefore did not undertake cost collection or analysis in this trial.

The correlation results for depressive symptoms and number of gaming sessions demonstrated that mental health outcomes were moving in the desired direction during the AVRG intervention. While the feasibility trial was not powered for detecting between-group differences, both PHQ-9 and DASS Stress scores decreased significantly between baseline and after the AVRG intervention, with almost all mental health measures collected during the trial showing changes indicating improved mental health.

Additionally, the significant dose–response relationship observed between reduced depressive symptoms and both a high number of gaming sessions over 30 minutes and more total gaming sessions performed suggests a potential causal link.

This dose response is echoed in the majority of the evidence where higher levels of traditional PA are associated with lower depressive symptoms [9] and even lower doses of PA—< 150 minutes of light to moderate-intensity PA—have been connected to reduced incidence of depression [47]. These results corroborate the limited research on AVRG/exergaming and mental health specifically in young men, with other studies showing improved anxiety [48], mental wellbeing and happiness [49].

Therefore, with the antidepressant effects of exercise well-established [10], these preliminary findings demonstrate the potential of AVRG to increase PA and mental health in the target population and that AVRG should be explored further in a fully-powered trial.

The significant increase in PA engagement over the eight-week intervention period as demonstrated in the gaming adherence data in addition to the self-reported IPAQ, suggests that AVRG could either directly increase PA and/or inspire other physically active lifestyle behaviours in this target population. These results are similar to the VR-based training intervention by Maden et al. [48], who also found that young adult male participants’ weekly PA level increased significantly (*p* = 0.032) compared to the control group. This is particularly encouraging in the context of the COVID-19 pandemic, as although participants were reportedly inactive prior to the intervention, even if they were interested in using local recreational or fitness facilities, this was not possible, due to lockdowns, social distancing and closure of many facilities.

In terms of PA behaviour change, with participants moving from contemplation to the preparation stage in readiness to change PA, there is a good indication this could have continued into a more consolidated action stage if the trial was 12 weeks, for example, which is endorsed by the body of evidence in health behaviour change interventions [50, 51].

Young men were proven to be a more difficult-to-reach population and less receptive than other populations to the online PA options available during the COVID-19 pandemic in a multi-country, cross-sectional analysis of PA, mental health and wellbeing during the pandemic [13]. The authors found engagement in online PA increased rapidly in women, however, males and younger adults reported more negative changes in exercise behaviour and, thus, were not sufficiently engaged by online PA options. However, this AVRG trial shows the potential to engage young men more successfully in this mode of PA and potentially improve mental health, through exercise engagement and, importantly, adherence [11].

The increase in quality-of-life scores on the SF-36 in the categories of energy/fatigue (vitality) and role functioning/emotional both demonstrate enhancements in mental health and are consistent with the improvements in mental health scores as observed in the PHQ-9 and DASS-21. These SF-36 and mental health outcomes combined indicate the intervention had positively affected mental health, and this corroborates the body of evidence on the potential for PA to improve both physical and mental health [11].

Although the changes in sleep were not statistically significant, the scores did indicate sleep was moving in a favourable direction. However, sleep is a complex and multi-factorial lifestyle behaviour, affected by mental health and likely also by COVID-19 during the trial and as such, it could be expected to take longer than eight weeks to show significant change in this case [52].

The increase in overall PA, with 50% of participants meeting National PA Guidelines [21] at the end of the intervention—compared to none of them at baseline—was achieved via the AVRG as well as engagement in other structured and non-structured PA. This demonstrated the flow-on effect of engaging in specified PA into other physically active behaviours, such as walking to work and taking up running. These findings support the evidence demonstrating the potential for PA to enhance other healthy lifestyle behaviours [53–55]. In addition, the decrease in sedentary gaming time and increase in active gaming and overall physical activity, it seems likely that participants swapped some non-active gaming time for active gaming, demonstrating potential for this approach to be useful as a simple, appealing lifestyle behaviour replacement to promote physical activity in this demographic.

There were several limitations to note with this investigation. Due to the desirability of the trial activities, there is the possibility of participants misrepresenting information during self-screening. This was regulated in our trial via the in-person phone call to verify the details on their screening and confirm eligibility, and also due to the fact that participants were not aware of the PHQ-9 inclusion criteria. A future, larger RCT in this area could consider screening by a psychologist to assess depressive symptoms. The potential for social desirability to influence outcomes was also considered. This was mitigated through the decision to administer all outcome measures via self-reported survey due to the evidence that in-person, interviewer-assisted methods, in particular for youth with regards to mental health, results in participants reporting lower psychological distress and higher wellbeing compared with the self-reported methods [56]. In fact, this trial demonstrates that although lacking in the control of in-person processes, a fully-online, automated trial process can be useful in the context of the recognised challenges in recruiting young people for face-to-face research interviews, particularly those dealing with sensitive subjects such as health, which can cause increased anxiety when talking to an unfamiliar adult [57].

All participants were located in urban areas of cities in several states across Australia. As this trial was conducted during the COVID-19 pandemic, frequent lockdowns during the trial meant the experience of being in an urban location at the time was atypical, and more akin to being in a remote or regional location without access to many options for recreation or PA. Future trials may examine the potential for AVRG to increase PA in various geographic settings, different populations and the opportunity for scalability.

Social aspects of the AVRG activity, such as playing with friends online or in person, were not quantified in this trial. Conducting research into the social components and effects of games played in online virtual environments with known and unknown people is challenging to monitor, randomise and control. However, research into the types of AVRGs that increase adherence with a particular focus on social aspects could enhance the effectiveness of this mode of PA in addressing depressive symptoms, particularly in contexts of restricted in-person contact and for individuals in remote and regional settings. The social isolation and loneliness of COVID-19 fuelled anxiety and depression, resulting in a global worsening of mental health [58], and the evidence supports social contact to enhance mental health [59, 60] and the benefits of engaging in PA with others, include an increase in accountability, fun and competition, which enhance motivation, adherence and health behaviour change [61]. Future studies could investigate social influence by recruiting groups to play AVRGs on the same server or testing a new initiative by the Institute of Virtual Reality Health and Exercise encompassing artificial intelligence to produce a fun, responsive exercise experience in a social multiplayer (four people) environment: Pacebreaker: AI-perfected exercise [62].

The COVID-19 delivery delays experienced affected the flow of trial procedures. The REDCap database was established for equipment to be delivered and set up within a week of participants completing baseline assessments for the active gaming period. The delays extended this period to up to three weeks for some participants.

In addition, motivating trial participants to complete outcome measures can prove challenging, especially when there is no incentive offered [59]. Of those remaining in the trial, 50% (*n* = 14) completed outcome assessments within two weeks, and a further 10 participants completed them within three and a half weeks. However, the participant who completed the outcome surveys four and a half weeks later and certainly the one who completed them 12 weeks post-intervention could feasibly have had any number of influencing factors on their mental health, physical health, and PA status; thus, the impact of the intervention may have diminished by that point. These outcome surveys collected the secondary outcome data, and as these ‘late completers’ represented a small proportion (7%) of the population who completed data collection (*n* = 28), this time lag is not estimated to have significantly impacted the overall results of the trial, because the primary outcomes were more focused on feasibility rather than being powered to detect change in the secondary outcomes.

A cost effectiveness analysis was not included in this study due to several factors including concern about participant burden, lack of access to resources during COVID and limited staffing during this time. Future fully powered studies should consider including EQ5D or similar tools to allow for a cost-effectiveness analysis to be undertaken.

Some of the RCT data collection procedures, such as wearing the HR monitor and starting the external app, were identified as barriers to engagement with the AVRG. It is possible participants would have engaged in or tracked more gaming without these trial-related procedures, possibly improving the outcomes for gaming adherence, PA levels and mental health. This is estimated to have influenced primary and secondary outcomes, but as a feasibility trial, it only adds to the novel investigation of procedures that facilitate or create barriers for desired PA behaviours to inform a fully powered RCT in this area.

Future AVRG trials can benefit from technological advances that were rapidly emerging during and since this study. For example, more sophisticated in-device activity tracking is now available within the Oculus Quest and similar VR gaming systems, which negates the need for additional external apps or monitoring devices. This reduces barriers to participation and eliminates trial procedures that would not typically occur in real-world gaming, thereby allowing pragmatic trials to more closely reflect real-life use.

COVID-19 affected all participants’ lifestyles during the trial. Further, although the WL group contracted COVID-19 mostly during the trial and may have had more time at home to play. In addition, the timing of the trial meant many of the WL participants were in the intervention phase over the December-January Summer holiday period in Australia, also potentially leading to more recreational time, especially for the majority who were undertaking tertiary education either full or part-time. This is not estimated to have had a major impact on the trial, as although the WL reported more COVID-19 cases than the active group, the WL group tracked more gaming and the data showed a similar pattern in both groups of more gaming in the first two weeks in all participants with a gradual decrease in most cases for the remainder of the intervention. It is also possible some participants in the WL group were more consistent with using the tracking devices, rather than that they actually did more gaming. The significant difference in household size observed between groups, was not expected to influence engagement with the intervention or trial outcomes.

Although the addition of a daily activity tracker (for example FitBit) may have given a more accurate representation of total PA alongside the IPAQ, this was not feasible due to trial budget. In addition, it would have increased the burden on participants to comply with extra trial devices alongside those already required to track active gaming. A 24-hour tracking device, in a non-clinical, non-supervised, pragmatic trial, would likely have resulted in similar inconsistencies in participant compliance to the tracking devices used. Fortunately, future trials may take advantage of the technological advances in both gaming and PA tracking.

VR gaming has been gaining research popularity in clinical populations and rehabilitation for both physical and mental health. However, using AVRG purely for increasing PA in physically inactive populations to enhance mental health is a novel area of investigation. In addition, using digital gaming enhanced accessibility during COVID-19 lockdowns and is also popular and perceived to be fun by the target population of young men [12]. However, this trial also investigated the novel prospect of influencing behaviour and improving health in those already engaged in many hours of sedentary gaming by swapping some time for active gaming. This does not represent a great barrier to increasing PA in this demographic, and, in fact, this demonstrates a strong case for feasibility. The acceptability of the mode of PA to the target population is key to ensure adherence long enough to increase PA and improve mental health and to continue this health behaviour long term to prevent the return of depressive symptoms.

Most studies on AVRG/exergaming have been exploratory, conducted in clinical or laboratory settings, with scheduled, supervised sessions. As such, there is little existing evidence of a dose response or investigating a relationship between the amount of gaming and changes in mental health, particularly in settings that reflect how AVRG is used in the real world (e.g., at home). This AVRG trial shows the real-world relevance through pragmatic research and the potential scalability, application, and the potential for greater reach demonstrated.

More in-depth evidence into the effect of the environment and contexts for AVRG trials would also enhance application in real-world settings, as suggested by other researchers in this area [63]. Future studies could evaluate the difference in the effects of laboratory or clinical settings and pragmatic settings and investigate the barriers and facilitators in participants’ own environments and strategies to decrease the former and increase the latter and the effects of using contextual cues to enhance health behaviours and habits.

Participants in this RCT were rated as affected by mild to moderate depressive symptoms on screening assessments and reported they were physically inactive prior to commencement of the intervention, and these young men all found AVRG an appealing activity to try. At the end of the trial, most were still engaged in AVRG, and many had added other forms of PA and health behaviours to their lifestyles as well. Additionally, not only did their mental health improve as a group, but those who engaged with more gaming, experienced greater improvement.

Studies investigating the types of games effective for meeting PA intensity and duration prescriptions that promote long-term adherence in the same way as popular group fitness in-person programs, such as F45 or CrossFit, would be particularly useful to strengthen evidence for the clinical prescription of AVRG for physical and mental health. Peloton is an example of an online program that achieved a ‘cult-like following’ and adherence enhanced by gamification, conformism and convenience [64]. However, this program requires a bulky, expensive exercise bike or treadmill equipment. AVRG has the advantages of more convenient equipment, a highly immersive user experience and the extra fun factor where the activities feel a lot less like ‘exercise’ than cycling or running, which is beneficial for those resistant to traditional forms of exercise.

The games most likely to be effective for adherence would be commercially popular rather than those designed and built for a trial, as the latter usually cannot compete with the quality expected from the target population. It would be interesting to evaluate, compare and contrast the following types of VR games for creating active habits and building substantial fitness levels and hence, enhancing mental health:

physically active competitive esports, Active recreational games (such as those used in this trial), and fitness games (simulated fitness class or personal training).

Evidence on AVRG for achieving fitness levels comparable to in-person programs could help combat the stigma that video games are unhealthy and remove a barrier to PA for many young men who may find this form of exercise highly acceptable.

This trial demonstrated that an AVRG intervention is feasible and acceptable to increase PA in a population of young men with mild to moderate depressive symptoms. While not powered to detect between-group differences, almost all measures of mental health improved over time with the AVRG intervention, demonstrating a potentially beneficial intervention. AVRG also helped motivate continued PA and other continued healthy lifestyle behaviours outside of the gaming sessions alone. Future, fully powered RCTs will be required to confirm these findings.

## Electronic supplementary material

Below is the link to the electronic supplementary material.

Supplementary Material 1

## Data Availability

Data is provided within the manuscript, in the appendices.

## Appendix A–Trial outcomes

### Primary outcomes

- Recruitment rate; interest in participating in the trial, identification of appropriate recruitment strategies and appropriateness of eligibility criteria - was calculated in two ways. First, as the percentage of individuals who were successfully enrolled out of *those who were screened as potentially eligible* from the screening questionnaire (the number of participants who were randomised from those who were eligible to join) and, second, the percentage of individuals who were successfully enrolled in the trial out of all who expressed interest in the study by completing the screening questionnaire (the number of participants who were randomised from all those who were interested in joining the study). These two outcomes combined report the interest in participating in the trial, appropriateness of recruitment strategies and eligibility criteria.
- Engagement and adherence; compliance with activity - was calculated as the number of participants who meet the recommendation for a minimum of three 30-minute sessions per week and for how many weeks they achieved this level, measured via activity-tracking app screenshots uploaded by participants at endpoint data collection.
- Retention was calculated as the percentage of participants to complete both the baseline and follow-up outcome measures.
- Safety was reported via weekly health check surveys.
- Appropriateness of the outcome measures was assessed through compliance with data collection. This was calculated by completing weekly health check surveys and uploading screenshots of tracked game playing to the REDCap database.

## Secondary outcomes

- Changes in depressive symptoms or mood via the PHQ-9 (Kroenke, 2020) and DASS-21 (Lovibond, 1995).
- Health-related quality of life via the Short Form-36 (SF-36) (McHorney et al., 1993).
- Exercise motivation via the Exercise Motivation Inventory-2 (EMI-2) (Markland & Ingledew, 1997).
- PA level via the IPAQ (The IPAQ Group, 2005).
- Sleep quality via the Pittsburgh Sleep Quality Index (PSQI) (Buysse et al., 1989).
- Readiness to change PA behaviour via one-question, previously validated format in line with the stages in the Transtheoretical Model of Change (TTM) (Cancer Prevention Research Center, 2020) as follows:.

Readiness for change outcome measure (for baseline, endpoint and follow-up surveys).

## Your Physical Activity (PA) level

The following questionnaire is a simple way of measuring the amount of PA you have done over the past week. It is strictly confidential so try to answer the question as honestly as you can.

**Table:**

REGULAR PA RELATES TO:	
Exercise	e.g. weight training, aerobics for 2–3 times per week; jogging or hill-walking for at least 2 hours/once per week
*Or* **Sport**	e.g. golf, hockey, football, netball, athletics, swimming for 2–3 times per week
*or* **General**	e.g. walking, mowing the lawn, vacuuming, washing the car accumulating to at least 30 minutes/4–5 times per week

.

Please read through all statements listed below and select the **ONE** statement that best describes your PA over the last 6 months.

1. I am not regularly physically active and do not intend to be in the next 6 months.
2. I am not regularly physically active but am thinking about starting to do so in the next 6 months.
3. I do some PA but not enough to meet the description of regular PA given above.
4. I am regularly physically active but only began in the last 6 months.
5. I am regularly physically active and have been so for longer than 6 months.

## Appendix B Active virtual reality gaming trial participant information form

### Participant information sheet—general (extended)

#### NICM health research institute

Project title: A randomised feasibility study assessing an Active Virtual Reality Gaming intervention to promote PA and mood in young men with mild–moderate depression.

## Project summary

You are invited to participate in a research study being conducted by Dr Mike Armour, Mrs Fiona Hargraves, (PhD Candidate) from NICM Health Research Institute, Western Sydney University, Australia; Dr Freya MacMillan, Dr Emma George and Dr Sandra Garrido from Western Sydney University, Australia; Dr Joseph Firth from The University of Manchester, England; and Professor Kerry Sherman, Macquarie University, Australia.

Young adulthood can be a busy and stressful time with many lifestyle changes. PA is known to be beneficial for stress, anxiety and improving mental health but it can also be difficult to find the time and motivation to exercise, especially with low mood or when feeling stressed. In addition, the recent COVID-19 pandemic has limited access to many community recreation and fitness centres and opportunities for PA have declined. Meanwhile, there is a rising interest in e-health and digital strategies for increasing PA.

We are interested in investigating whether playing active virtual reality games on the Oculus Quest 2 device can promote PA adherence and enhance mood in young Australian men. This information will help us better understand the role of alternative modes for involvement in PA, rather than attending a fitness centre in person and whether active virtual reality gaming (AVRG) is potentially an acceptable and feasible strategy to help guide future research and practice in accessible PA interventions for mental health.

You do not need to be currently undertaking any structured PA or gaming to participate.

How is the study being paid for? This study is part of a PhD funded by the Blackmores Institute.

## What will I be asked to do?

This trial is open to participants Australia wide.

To be eligible to be part of this trial you need to:

- be a male aged between 18 and 29 years
- willing and physically able to participate in active VR gaming
- have reliable Wi-Fi internet connection, access to a smartphone to for screening and outcome apps, willing to use a Facebook account to log into an Oculus Quest 2 device
- have a safe and clear 2 × 2 m space at their place of residence in which to operate the gaming device
- willing to provide informed consent and adhere to the protocol
- not currently be taking any pharmaceutical medication (such as an antidepressant prescribed by your doctor) or any herbal medicines (like St John’s Wort) that are designed to improve your mood.

You will be asked to fill in an online survey to determine eligibility for the trial. You will be asked about your background (such as your age, which state you live in, employment status), current lifestyle (PA, sleep, habits), experience with exercise, preferences for exercise involvement, mood and stress (mental health).

If eligible, you will be randomised into one of two groups, who are both involved in the same gaming intervention (one group has a delayed start) and undertake baseline measures consisting of online surveys after which you will receive an Oculus Quest 2 device on loan to use at home through your Facebook account and receive orientation for use. You will participate in an eight-week program of AVRG, in which you can choose to play any or all of three provided games: Pistol Whip, Thrill of the Fight and Beat Sabre/Audio Trip. While playing on the Oculus Quest, you will wear a provided heart rate monitor linked to the VR Exercise app to track usage and physical exertion.

After eight weeks, you will return the device to us and complete online surveys of outcome measures. And then a short survey online four weeks after that to follow up.

## How much of my time will I need to give?

Participation in the study will involve:

- initial baseline measures: around 30 minutes
- AVRG for 8 weeks: at least three 30-minute sessions each week
- outcome measures: around 30 minutes
- (four-week follow-up survey: 10 minutes)
- (post-trial interview: 30 minutes)

.

## What benefits will I, and/or the broader community, receive for participating?

There is existing evidence that PA can enhance both mental and physical health and you also get the opportunity to learn and participate in some popular, fun virtual reality gams with excellent user reviews. The findings from this trial may be used to direct future research on PA via AVRG and mental health in young men.

## Will the study involve any risk or discomfort for me? If so, what will be done to rectify it?

We do not foresee any major risk to you from participating in this study. For online surveys, we do not use cookies or collect your IP address and all information collected is stored in the REDCap clinical trials software system as a number you will be allocated once randomised. There are risks involved in participating in PA and we will provide safety recommendations aligned with the use of the gaming equipment. We recognise that some people feel uncomfortable when talking about matters relating to physical and mental health.

If any of the topics raised in this trial cause distress, or for more information about stress, mood or mental health please consider contacting one of the support services listed below:.

Lifeline: 13 11 14 (https://www.lifeline.org.au/) Beyond Blue: 1300 224 636 Suicide Call Back Service: 1300 659 467 The Suicide Call Back Service provides immediate support to anyone feeling suicidal. In addition, they can provide ongoing support through up to six 50-minute telephone counselling sessions that will provide you with longer-term support. The Suicide Call Back Service also offers online counselling.

## How do you intend to publish or disseminate the results?

It is anticipated that the results of this research project will be published and/or presented in a variety of forums. In any publication and/or presentation, information will be provided in such a way that the participant cannot be identified, via summaries of what we found, rather than individual data.

## Will the data and information that I have provided be disposed of?

Please be assured that only the researchers will have access to the raw data you provide. However, your data may be used in other related projects for an extended period of time, such as helping inform future clinical trial design on PA and mental health interventions.

## Can I withdraw from the study?

Participation is entirely voluntary, and you are not obliged to be involved. If you do participate you can withdraw at any time. We will retain data collected up to the point of withdrawal for analysis because this is important information in a feasibility study. Whatever your decision, it will not affect your relationship to anyone involved in this study.

## Can I tell other people about the study?

Yes, you can tell other people about the study by providing them the link to this information sheet. If they would like more information, they can then contact Dr Armour to discuss their participation in the research project.

## What if I require further information?

Please contact Dr Mike Armour should you wish to discuss the research further before deciding whether or not to participate.

Dr Mike Armour, Chief Investigator, email m.armour@westernsydney.edu.au.

## What if I have a complaint?

If you have any complaints or reservations about the ethical conduct of this research, you may contact the Ethics Committee through Research Engagement, Development and Innovation (REDI) on Tel +61 2 4736 0229 or email humanethics@westernsydney.edu.au.

Any issues you raise will be treated in confidence and investigated fully, and you will be informed of the outcome.

This study has been approved by the Western Sydney University Human Research Ethics Committee. The approval number is H14118.

## What will happen with my information if I agree to it being used in projects other than this one?

Thank you for considering being a participant in a university research project. The researchers are asking that you agree to supply your information (data) for use in this project and to also agree to allow the data to potentially be used in future research projects.

This request is in line with current university and government policy that encourages the re-use of data once it has been collected. Collecting information for research can be an inconvenience or burden for participants and has significant costs associated with it. Sharing your data with other researchers gives potential for others to reflect on the data and its findings, to re-use it with new insight, and increase understanding in this research area.

You have been asked to agree to extended consent.

## Extended consent

When you agree to extended consent, it means that you agree that your data, as part of a larger dataset (the information collected for this project) can be re-used in projects that are

- an extension of this project
- closely related to this project
- in the same general area of this research.

The researchers will allow this data to be used by our research team at NICM Health Research Institute to inform future research in this area.

To enable this re-use, your data will be held at the university in its data repository and managed under a Data Management Plan. The stored data available for re-use *will not* have information in it that makes you identifiable. The re-use of the data will only be allowed after an ethics committee has agreed that the new use of the data meets the requirements of ethics review.

The researchers want to keep the data for 1*5 years* for possible re-use. After this time the data will be securely destroyed.

You are welcome to discuss these issues further with the researchers before deciding if you agree. You can also find more information about the re-use of data in research in the National Statement on Ethical Conduct in Human Research—see Sections 2.2.14—2.2.18.

https://www.nhmrc.gov.au/about-us/publications/national-statement-ethical-conduct-human-research-2007-updated-2018.

## Appendix C Active virtual reality gaming trial consent form

### Consent Form—General (Extended)—NICM Health Research Institute

Project title: A randomised feasibility study assessing the effect of an active virtual reality gaming intervention on PA and mood in young men with mild–moderate depression. This study has been approved by the Human Research Ethics Committee at Western Sydney University. The ethics reference number is: H14118.

I hereby consent to participate in the above-named research project. I acknowledge that: • I have read the participant information sheet (or where appropriate, have had it read to me) and have been given the opportunity to discuss the information and my involvement in the project with the researcher/s.

• The procedures required for the project and the time involved have been explained to me, and any questions I have about the project have been answered to my satisfaction.

**I consent to:**.

☐ *Participating in surveys and outcome assessments in digital survey form*.

☐ *Participating in active virtual reality gaming on the Oculus Quest 2 device linked to a personal Facebook account*.

☐ *Tracking gaming sessions with the VR Exercise app and a heart rate monitor*.

I consent for my data and information provided to be used in this project and other related projects for an extended period of time.

I understand that my involvement is confidential and that the information gained during the study may be published and stored for other research use but no information about me will be used in any way that reveals my identity.

I understand that my participation in this study will have no effect on my relationship with the researcher/s, and any organisations involved, now or in the future. I understand that I will be unable to withdraw my data and information from this project. *[As a feasibility trial, discontinued participation provides important information on engagement and adherence.]*.

☐ *I would like to be provided with a summary of results from this study via email once analysed and published.*

**Signed:**.

**Name:**.

**Date:**.

**What if I have a complaint?**

If you have any complaints or reservations about the ethical conduct of this research, you may contact the Ethics Committee through Research Engagement, Development and Innovation (REDI) on Tel +61 247,360,229 or email http://humanethics@westernsydney.edu.au.

Any issues you raise will be treated in confidence and investigated fully, and you will be informed of the outcome.

## Appendix D Ethics approval for the active virtual reality gaming trial

### November 2020

Doctor Mike Armour.

NICM Health Research Institute.

Dear Mike,.

HREC Approval Number: H14118 Risk Rating: Moderate.

Human Research Ethics Committee.

I am pleased to advise the above research project meets the requirements of the National Statement on Ethical Conduct in Human Research 2007 (Updated 2018).

Ethical approval for this project has been granted by the Western Sydney University Human Research Ethics Committee. This HREC is constituted and operates in accordance with the National Statement on Ethical Conduct in Human Research 2007 (Updated 2018).

Approval of this project is valid from 27 November 2020 until 27 July 2021. This protocol covers the following researchers:.

Mike Armour, Fiona Hargraves, Sandra Garrido, Joe Firth, Kerry Sherman, Freya Macmillan, Emma George Summary of Conditions of Approval.

1. A progress report will be due annually on the anniversary of the approval date.
2. A final report will be due at the expiration of the approval period.
3. Any amendments to the project must be approved by the Human Research Ethics Committee prior to being implemented. Amendments must be requested using the HREC Amendment Request Form.
4. Any serious or unexpected adverse events on participants must be reported to the Human Research Ethics Committee via the Human Ethics Officer as a matter of priority.
5. Any unforeseen events that might affect continued ethical acceptability of the project should also be reported to the Committee as a matter of priority.
6. Consent forms are to be retained within the archives of the School or Research Institute and made available to the Committee upon request.
7. Approval is only valid while you hold a position or are enrolled at Western Sydney University. You will need to transfer your project or seek fresh ethics approval from your new institution if you leave Western Sydney University.
8. Project specific conditions:.

There are no specific conditions applicable.

Please quote the registration number and title as indicated above in the subject line on all future correspondence related to this project. All correspondence should be sent to humanethics@westernsydney.edu.au as this email address is closely monitored.

Yours sincerely.

Professor Brett Bowden.

Presiding Member, Western Sydney University Human Research Ethics Committee.

## Appendix E – Trial technology

### Oculus Quest 2 (virtual reality headset)

The device used in this trial was the 64 GB Oculus Quest 2 wireless VR headset released by Facebook Technologies in October 2020. This device requires no additional console and features tracking in which sensors translate movements into a room-scale VR environment. It also includes hand-held touch controllers that allow hands and gestures to appear in VR with realistic precision. The Oculus Quest requires users to have a Facebook (now Meta) account to use. Other features that enhance user experience include an adjustable headband and eye lenses and an attachment to facilitate wearing glasses with the headset. Another reason this device was selected for the trial was the safety features not available in other VR headsets at the time, such as a virtual ‘safety net’, which appears in the user’s field of vision if they approach pre-set boundaries to the real physical space and an external camera that displays the real environment this boundary is breached, preventing collisions with obstacles.

## Oculus move app

Participants were required to set up and activate the Oculus Move app, which is a feature provided by the Oculus Quest 2 software. The app requests input of gender, age, height, and weight data as well as ‘move goals’ expressed in minutes and/or calories per day. Participants in this trial were instructed to set the move goals to 30 minutes and 50 calories per day. The Oculus Quest 2 tracks activity via the headset and hand controller movements and estimates energy expenditure based on user information provided; it also records minutes of active play. Although this is only an estimate and not an accurate measure of PA intensity due to calculations used, it was considered a corroborating measure to compare to the more accurate external tracking data detailed below to verify if participants had connected to this external tracking method for each session.

The minutes counted by the Oculus Move app are represented in a pie chart and do not provide an accurate number of minutes as data output; they only reflect progression towards the set goal of 30 minutes.

## Activity tracking—VR Exercise smartphone app and heart rate monitor

Participants were provided with a Polar H7 chest strap HR monitor to wear and connect via Bluetooth to an external smartphone app each time they engaged in AVRG. The app selected, the VR Health Exercise Tracker app, was created by the Virtual Reality Institute of Health and Exercise is available on both Android and iOS platforms, and tracks minutes, HR and estimated caloric expenditure for the selected game. The VR Institute of Health and Exercise has devised equivalent activity and calorie ratings for commercially popular VR games by measuring oxygen consumption with a metabolic cart, as follows:

- resting: 1–2 calories per minute
- walking: 2–4 calories per minute
- elliptical: 4–6 calories per minute
- tennis: 6–8 calories per minute
- rowing: 8–10 calories per minute
- biking: 10–12 calories per minute.

The ratings are based on an individual weighing 60 kg with at least a moderate amount of familiarity and skill in the games [20].

## Active virtual reality games

The three commercially popular active VR games for this trial were chosen according to PA equivalent ratings of tennis or higher, as per the Virtual Reality Institute of Health and Exercise [20].

Thrill of the Fight: a boxing simulation, including training and boxing matches—rated 8–10 calories per minute or equivalent to rowing.

Pistol Whip: a shooting simulation, including travelling through various environments, targeting non-realistic humanoids and dodging sites and bullets—rated 6–8 calories per minute or equivalent to tennis.

Beat Sabre: a rhythm game requiring speed, coordination, and accuracy to strike oncoming targets in time with music while evading moving objects—rated 6–8 calories per minute or equivalent to tennis.

VR gaming is considered safe. However, contraindications and cautions include recent injuries or ongoing musculoskeletal issues with the neck and upper extremities, serious vision or balance issues and photosensitivity epilepsy.

Participants were recommended to engage in a minimum dose of 90 minutes per week (three 30-minute sessions) of AVRG, with no upper limit set. This aligns with the *Australian PA Guidelines*’ 75–150 minutes of vigorous-intensity activity [65]. Participants were encouraged to accumulate this across several sessions per week.

## Appendix F – Trial flow chart

Note: AE = Adverse Event. Post-trial in-depth interviews will be reported separately.

## Appendix G – Trial results; additional secondary results

### Outcome variables - mean and standard deviation by intervention

**Table:**

	**Mean (SD)**.			
	**Active N = 14**.		**Waitlist N = 14**.	
	**Baseline**.	**Endpoint**.	**Baseline**.	**Endpoint**.
PHQ-9.	9.9 (2.6).	7 (5.1).	7.8 (3.8).	7.6 (4.8).
DASS-21 Anxiety.	7.7 (4.8).	5.7 (5).	4.5 (4.6). *N* = 13.	6.4 (5.6).
DASS-21 Depression.	11.4 (5.5).	7.3 (6.5).	11.3 (7.2).	10.9 (7.4). *N* = 13.
DASS-21 Stress.	14.8 (9.6). *N* = 13.	11.1 (9).	10.2 (8). *N* = 13.	11.3 (7).
EMI-2 Stress management.	2.3 (1.2).	2.4 (1.5). *N* = 13.	2 (1.5).	1.9 (1.1).
EMI-2 Revitalisation.	2 (1.6).	2.4 (1.4). *N* = 13.	2.1 (1.1).	2 (1.1).
EMI-2 Enjoyment.	2 (1.5).	2.3 (1.4). *N* = 12.	1.9 (1.5).	2.1 (1.6).
EMI-2 Challenge.	2.5 (1.8).	2.4 (1.6). *N* = 13.	1.8 (1.3).	2.2 (1.6). *N* = 13.
EMI-2 Social recognition.	1.1 (1.4). *N* = 13.	1.5 (1.3). *N* = 13.	1.6 (1.3).	1.2 (1.5). *N* = 13.
EMI-2 Affiliation.	1.2 (1.5). *N* = 13.	1.4 (1.2). *N* = 13.	1.7 (1.6).	1.9 (1.5).
EMI-2 Competition.	1.7 (1.5). *N* = 13.	1.4 (1.4). *N* = 13.	1.6 (1.6).	1.6 (1.4).
EMI-2 Health pressures.	0.6 (0.8).	0.8 (0.9). *N* = 13.	0.8 (0.8).	0.7 (1.1). *N* = 13.
EMI-2 Ill-health avoidance.	2.8 (1.8).	3.4 (1.1). *N* = 13.	2.8 (1.3).	2.7 (1.1).
EMI-2 Positive health.	3.5 (1.5).	3.7 (1). *N* = 12.	3.8 (0.9).	3.6 (0.9).
EMI-2 Weight management.	3.6 (1.7).	3.6 (1.4). *N* = 13.	2.7 (1.8).	2.7 (1.8).
EMI-2 Appearance.	3.1 (1.3).	3.3 (1). *N* = 13.	2.8 (1.1).	2.7 (1.2).
EMI-2 Strength & endurance.	3.5 (1.8).	3.4 (1.6). *N* = 12.	3.6 (1.1).	3.3 (1.2).
EMI-2 Nimbleness.	2.8 (1.8).	3 (1.3). *N* = 13.	2.7 (1).	2.6 (1.3).
State of change.	2.6 (1.2).	3.1 (1.1).	2.4 (0.6).	2.5 (0.8).
PSQI.	8.1 (2.5).	8.3 (2.9).	6.9 (3).	6.9 (3.5).
SF-36 Physical functioning.	87.9 (15.8).	92.1 (14.5).	95 (8.5).	95.4 (7.5).
SF-36 Role functioning/physical.	82.1 (24.9).	87.5 (27.3).	76.8 (33.2).	82.1 (31.7).
SF-36 Role functioning/emotional.	26.2 (26.7).	69.2 (39.6). *N* = 13.	31 (33.2).	42.9 (40.1).
SF-36 Energy/fatigue.	37.5 (13).	45.2 (15.2).	41.8 (21.1).	36.1 (22).
SF-36 Emotional wellbeing.	60.3 (15.8).	68 (14.8).	61.4 (17.5).	62.6 (13.2).
SF-36 Social functioning.	73.2 (23.4).	67.9 (28.5).	70.5 (20.6).	76.8 (20.1).
SF-36 Pain.	83.4 (19.8).	78.9 (18.5).	87.7 (12.1).	82.1 (22.9).
SF-36 General health.	56.8 (20.3).	62.1 (18.9).	60.7 (17.6).	63.6 (19.9).
IPAQ.	1.4 (0.6).	2.1 (0.9).	1.3 (0.5).	1.8 (0.6).

## WL pre–post

**Table:**

**Analysis**.	**Pre-minus post-estimate of mean difference (95% CI)**.	**P**.
PHQ-9.	0.14 (‒1.47 to 1.76).	0.85.
DASS-21 Anxiety.	‒0.92 (‒3.87 to 2.02).	0.51.
DASS-21 Stress.	‒0.62 (‒3.98 to 2.75).	0.70.
EMI-2 Stress management.	0.13 (‒0.‒0.40 to 0.65).	0.61.
EMI-2 Revitalisation.	0.12 (‒0.28 to 0.52).	0.54.
EMI-2 Enjoyment.	‒0.16 (‒0.75 to 0.43).	0.57.
EMI-2 Challenge.	‒0.46 (‒0.91 to ‒0.02).	0.04.
EMI-2 Social recognition.	0.29 (‒0.27 to 0.85).	0.28.
EMI-2 Affiliation.	‒0.16 (‒0.71 to 0.39).	0.54.
EMI-2 Ill-health avoidance.	0.17 (‒0.36 to 0.69).	0.50.
EMI-2 Positive health.	0.17 (‒0.30 to 0.63).	0.45.
EMI-2 Strength & endurance.	0.36 (0.00 to 0.71).	0.0498.
EMI-2 Nimbleness.	0.10 (‒0.43 to 0.62).	0.70.
Stage of change.	‒0.14 (‒0.74 to 0.45).	0.61.
PSQI.	0 (‒1.24 to 1.24).	1.00.
SF-36 Energy/fatigue.	5.71 (‒1.70 to 13.13).	0.12.
SF-36 Emotional wellbeing.	‒1.14 (‒8.01 to 5.72).	0.72.
SF-36 Pain.	5.54 (‒5.72 to 16.79).	0.31.
SF-36 General health.	‒2.86 (‒7.62 to 1.91).	0.22.
DASS-21 Depression^1^.	0 (‒4 to 6) (Estimate of median difference [95% CI]).	0.82.
EMI-2 Competition^1^.	0 (0 to 0.3).	0.99.
EMI-2 Health pressures^1^.	0 (‒0.7 to 1.0).	0.67.
EMI-2 Weight management^1^.	0 (‒0.5 to 1.3).	0.82.
EMI-2 Appearance^1^.	0 (‒0.5 to 0.5).	1.00.
SF-36 Physical functioning^1^.	0 (‒5 to 5).	1.00.
SF-36 Role functioning/physical^1^.	0 (‒25 to 0).	0.66.
SF-36 Role functioning/emotional^1^.	0 (‒33.3 to 0).	0.06.
SF-36 Social functioning^1^.	‒12.5 (‒12.5 to 0).	0.12.
IPAQ^1^.	‒1 (‒1 to 0).	0.09.

^1^ Normality assumption not met

## Mental health correlations

**Table:**

**Variables**.	**Correlation coefficient**.	**p**.
Week 9 PHQ-9 and number of sessions > 30 mins.	−0.47.	0.01.
Week 9 PHQ-9 and total number of sessions.	−0.57.	0.001.
Baseline stage of change and number of sessions > 30 mins^1^.	−0.07.	0.72.
Baseline stage of change and total number of sessions^1^.	−0.05.	0.80.
Week 9 DASS Anxiety and number of sessions > 30 mins.	−0.19.	0.34.
Week 9 DASS Anxiety and total number of sessions.	−0.22.	0.28.
Week 9 DASS Depression and number of sessions > 30 mins.	−0.43.	0.03.
Week 9 DASS Depression and total number of sessions.	−0.36.	0.07.
Week 9 DASS Stress and number of sessions > 30 mins.	−0.29.	0.14.
Week 9 DASS Stress and total number of sessions.	−0.25.	0.20.

^1^ Spearman’s correlation

## IPAQ outcomes

**Table:**

**Variable**.	**N**.	**Mean (SD)**.	**95% CI**.	**Median (IQR)***.	**Min to Max***.
Pre-IPAQ.	28.	1.6 (0.6).	1.38–1.82.	2 (1 to 2).	1 to 3.
Post-IPAQ.	28.	2.1 (0.8).	1.80–2.40.	2 (1 to 3).	1 to 3.

1 = low PA, 2 = moderate, 3 = high

## EMI outcomes

**Table:**

**Variable**.	**N**.	**Mean (SD)**.	**95% CI**.	**Median (IQR)**.	**Min to Max**.
**Pre-intervention**.					
EMI-2 Stress management.	28.	2.1 (1.1).	1.69–2.51.	2 (1.3 to 2.6).	0 to 4.5.
EMI-2 Revitalisation.	28.	2 (1.3).	1.52–2.48.	2.2 (0.7 to 3).	0 to 4.3.
EMI-2 Enjoyment.	28.	2.1 (1.5).	1.54–2.66.	2 (0.9 to 3.3).	0 to 4.5.
EMI-2 Challenge.	27.	2.4 (1.7).	1.76–3.04.	2 (1 to 4.3).	0 to 5.
EMI-2 Social recognition.	26.	1.2 (1.4).	0.66–1.74.	0.8 (0 to 1.8).	0 to 4.3.
EMI-2 Affiliation.	27.	1.6 (1.5).	1.03–2.17.	1.5 (0 to 3).	0 to 4.8.
EMI-2 Competition.	27.	1.6 (1.5).	1.03–2.17.	1.3 (0.3 to 3).	0 to 4.5.
EMI-2 Health pressures.	27.	0.7 (1.0).	0.32–1.08.	0 (0 to 1.3).	0 to 3.
EMI-2 Ill-health avoidance.	28.	2.7 (1.4).	2.18–3.22.	3 (1.7 to 3.7).	0 to 5.
EMI-2 Positive health.	28.	3.6 (1.2).	3.16–4.04.	3.5 (2.7 to 4.5).	0.7 to 5.
EMI-2 Weight management.	28.	3.1 (1.8).	2.43–3.77.	3.8 (1.9 to 4.8).	0 to 5.
EMI-2 Appearance.	28.	2.9 (1.3).	2.42–3.38.	3.3 (2 to 3.9).	0 to 5.
EMI-2 Strength & endurance.	28.	3.4 (1.5).	2.84–3.96.	3.8 (2.4 to 4.8).	0 to 5.
EMI-2 Nimbleness.	28.	2.7 (1.6).	2.11–3.29.	3 (1.3 to 4).	0 to 5.
**Post-intervention**.					
EMI-2 Stress management.	26.	2 (1.5).	1.42–2.58.	1.6 (0.8 to 2.8).	0 to 4.8.
EMI-2 Revitalisation.	27.	2.1 (1.3).	1.61–2.59.	2.3 (1 to 3.3).	0 to 4.3.
EMI-2 Enjoyment.	26.	2 (1.4).	1.46–2.54.	1.8 (0.8 to 3.5).	0 to 4.3.
EMI-2 Challenge.	26.	2 (1.5).	1.42–2.58.	1.8 (0.5 to 2.8).	0 to 5.
EMI-2 Social recognition.	27.	1.3 (1.3).	0.81–1.79.	1 (0.3 to 2.3).	0 to 4.3.
EMI-2 Affiliation.	27.	1.5 (1.6).	0.90–2.10.	0.8 (0.3 to 2.5).	0 to 5.
EMI-2 Competition.	27.	1.5 (1.6).	0.90–2.10.	0.8 (0 to 3.3).	0 to 4.8.
EMI-2 Health pressures.	27.	0.8 (1.0).	0.42–1.18.	0.3 (0 to 1.3).	0 to 3.3.
EMI-2 Ill-health avoidance.	27.	2.9 (1.2).	2.45–3.35.	3 (2 to 3.7).	0 to 5.
EMI-2 Positive health.	26.	3.6 (1.0).	3.22–3.98.	3.7 (3 to 4.3).	1.3 to 5.
EMI-2 Weight management.	27.	2.9 (1.6).	2.30–3.50.	3.3 (1.5 to 4).	0 to 5.
EMI-2 Appearance.	27.	2.8 (1.2).	2.35–3.25.	3 (1.8 to 3.8).	0.5 to 5.
EMI-2 Strength & endurance.	25.	3.1 (1.4).	2.55–3.65.	3.5 (2 to 4).	0 to 5.
EMI-2 Nimbleness.	27.	2.8 (1.2).	2.35–3.25.	3 (2 to 4).	0.3 to 5.

## Readiness to change outcomes

**Table:**

**Variable**.	**N**.	**Mean (SD)**.	**95% CI**.	**Median (IQR)**.	**Min to Max**.
Pre-SOC.	28.	2.5 (1).	2.13–2.87.	2 (2 to 3).	1 to 5.
Post-SOC.	28.	3.1 (1).	2.73–3.47.	3 (2 to 4).	1 to 5.

## SF-36 outcomes

**Table:**

**Variable**.	**N**.	**Mean (SD)**.	**95% CI**.
**Pre-intervention**.			
SF-36 Physical functioning.	28.	91.6 (12.7).	86.9–96.3.
SF-36 Role functioning/physical.	28.	82.1 (27.9).	71.77–92.43.
SF-36 Role functioning/emotional.	28.	34.5 (34.5).	21.72–47.28.
SF-36 Energy/fatigue.	28.	36.8 (17.8).	30.21–43.39.
SF-36 Emotional wellbeing.	28.	61.4 (14.3).	56.10–66.70.
SF-36 Social functioning.	28.	75 (21.5).	67.04–82.96.
SF-36 Pain.	28.	82.8 (21).	75.02–90.58.
SF-36 General health.	28.	60.2 (20.1).	52.75–67.65.
**Post-intervention**.			
SF-36 Physical functioning.	28.	93.9 (11.8).	89.53–98.27.
SF-36 Role functioning/physical.	28.	86.6 (29.3).	75.75–97.45.
SF-36 Role functioning/emotional.	27.	69.1 (38).	54.77–83.43.
SF-36 Energy/fatigue.	28.	47.4 (17.6).	40.88–53.92.
SF-36 Emotional wellbeing.	28.	67.7 (14.3).	62.40–73.00.
SF-36 Social functioning.	28.	71.4 (28.4).	60.88–81.92.
SF-36 Pain.	28.	81.8 (17.3).	75.39–88.21.
SF-36 General health.	28.	63.9 (18.6).	57.01–70.79.

## PSQI outcomes

**Table:**

**Variable**.	**N**.	**Mean (SD)**.	**95% CI**.	**Median (IQR)**.	**Min to Max**.
Pre-PSQI.	28.	7.5 (3).	6.39–8.61.	7 (5 to 9.5).	2 to 14.
Post-PSQI.	28.	7.1 (3.3).	5.88–8.32.	7 (5 to 9).	1 to 16.

## Appendix H – Trial results; participant lifestyle

### Baseline and post-intervention

**Table:**

**Lifestyle factors**.	**Baseline 1**. **n = 30**.	**Baseline 2**^**a**^. **n = 16**.	**Endpoint**. **n = 28**.
How important do you feel it is to lead an active lifestyle? (1 = not important, 10 = extremely important) Median (IQR).	7.5 (6,7,8,9,10).	7.5 (6.5–9).	8 (6,7,8,9,10).
How confident do you feel at the moment in being able to lead an active lifestyle? (1 = not confident, 10 = extremely confident) Median (IQR).	4 (4,5).	5.5 (4.5–6.5).	5 (5,6,7).
**Do any of these factors make it difficult to live an active lifestyle? (Select as many as apply)** ^**b**^ **n (%)**.	**n = 30**.	**n = 16**.	**n = 27**.
Lack of time.	9 (30).	11 (68.8).	20 (74.1).
Lack of motivation or willpower.	25 (83.3).	14 (87.5).	20 (74.1).
COVID-19 restrictions.	17 (56.7).	5 (31.3).	8 (29.6).
Knowing what to do (confusing/conflicting health information/advice).	6 (20).	6 (37.5).	7 (25.9).
Budget concerns.	8 (26.7).	3 (18.8).	6 (22.2).
Geography/logistics—access to recreational facilities.	6 (20).	3 (18.8).	5 (18.5).
Social factors—habits of those you spend time with (friends, family).	14 (46.7).	6 (37.5).	11 (40.7).
Other health issues.	1 (3.3).	1 (6.25).	2 (7.4).
Other (please specify) Participants identified issues related to work activities and schedule, and insecurities around weight and fitness.	4 (13.3).	0 (0.0).	1 (3.7).
None of the above.	0 (0.0).	0 (0.0).	0 (0.0).
**How long are you currently spending on non-active gaming each week? n (%) (Please specify platforms)**.	**n = 30**.	**n = 16**.	**n = 28**.
None.	1 (3.3).	0 (0).	0 (0.0).
<30 mins.	1 (3.3).	1 (6.25).	0 (0.0).
30–60 mins.	3 (10.0).	0 (0).	6 (21.4).
60–120 mins.	1 (3.3).	1 (6.25).	9 (32.1).
120–180 mins.	7 (23.3).	6 (37.5).	5 (17.9).
>180 mins.	17 (56.7).	8 (50).	8 (28.6).
**How long are you currently spending on active gaming each week? n (%) (Please specify platforms)**.	**n = 30**.	**n = 16**.	^**c**^.
None.	29 (96.7).	13 (81.3).	NA.
<30 mins.	1 (3.3).	2 (12.5)).	NA.
30–60 mins, 60–120 mins, 120–180 mins, > 180 mins.	0 (0).	0 (0).	NA.
No response.	0 (0).	1 (6.25).	NA.
**How much exercise or structured PA of moderate or greater intensity are you participating in per week? (e.g., gym, running, cycling. Moderate intensity = a bit of puffing, difficult to hold a conversation)** ^**d**^ **n (%)**.	**n = 30**.	^**e**^.	**n = 26**.
None.	25 (83.3).		8 (30.8).
<30 mins.	5 (16.7).		2 (7.7).
30–60 mins.	0 (0.0).		3 (11.5).
60–120 mins.	0 (0.0).		6 (23.1).
120–180 mins.	0 (0.0).		3 (11.5).
>180 mins.	0 (0.0).		4 (15.4).

^a^ Baseline 2 was completed by WL group only

^b^ Multiple selections were permitted; percentages total > 100

^c^ NA—not applicable. Participants were not asked about active gaming in the endpoint survey as they had most recently been enrolled in the AVRG trial, and that information was collected in the tracking data

^d^ Inclusion criteria for the trial were physical inactivity, < 30 mins per week non-structured/incidental PA

^e^ Participants were not asked about PA at baseline 2, as they were encouraged to change nothing in particular in their lifestyle during the WL period

## Four-week follow-up

**Table:**

**Lifestyle factors**.	**n = 15**. **n (%)**.
How important do you feel it is to lead an active lifestyle? (1 = not important, 10 = extremely important) Median (IQR).	8 (6,7,8,9,10).
How confident do you feel at the moment in being able to lead an active lifestyle? (1 = not confident, 10 = extremely confident) Median (IQR).	6.5 (4,5,6,7,8).
**Do any of these factors make it difficult to live an active lifestyle? (Select as many as apply)**^**b**^**n (%)**.	
Lack of time.	7 (46.7).
Lack of motivation or willpower.	11 (73.3).
COVID-19 restrictions.	1 (6.7).
Knowing what to do (confusing/conflicting health information/advice).	5 (33.3).
Budget concerns.	3 (20.0).
Geography/logistics—access to recreational facilities.	3 (20).
Social factors—habits of those you spend time with (friends, family).	7 (46.7).
Other health issues.	1 (6.7).
Other (please specify) Participants identified issues related to work activities and schedule, and insecurities around weight and fitness.	0 (0.0).
None of the above.	0 (0.0).
**Are you still participating in active Virtual Reality gaming?**	
No—and not interested in continuing.	4 (26.7).
No—but would continue if I had the device.	8 (53.3).
Yes—occasionally.	1 (6.7).
Yes—regularly.	2 (13.3).
**How long are you currently spending on non-active gaming each week?** **n (%) (Please specify platforms)**.	
None.	0 (0.0).
<30 mins.	2 (13.3).
30–60 mins.	3 (20.0).
60–120 mins.	3 (20.0).
120–180 mins.	3 (20.0).
>180 mins.	4 (26.7).
**How long are you currently spending on active gaming each week?** **n (%) (Please specify platforms)**.	
None.	11 (78.6).
30–60 mins.	2 (14.3).
>180 mins.	1 (7.1).
<30 mins 60–120 mins, 120–180 mins,.	0 (0.0).
**How much exercise or structured PA of moderate or greater intensity are you participating in per week? (e.g., gym, running, cycling. Moderate intensity = a bit of puffing, difficult to hold a conversation)** ^**d**^. **n (%)**.	
None.	5 (33.3).
<30 mins.	1 (6.7).
30–60 mins.	1 (6.7)).
60–120 mins.	3 (2.0).
120–180 mins.	1 (6.7).
>180 mins.	4 (26.7).

## Abbreviations

ACTRN: Australian and New Zealand Clinical Trials Registry registration number
AVRG: Active virtual reality gaming
CI: Confidence Interval
DASS-21: Depression, Anxiety and Stress Score − 21
EMI-2: Exercise Motivation Inventory-2
JMIR: Journal of Medical Internet Research
MD: Mean Difference
MET: Metabolic Equivalent Task
NA: Not Applicable
PA: PA
PAR-Q: Physical Activity Readiness Questionnaire
PHQ-9: Patient Health Questionnaire-9
PSQI: Pittsburgh Sleep Quality Index
RCT: Randomised controlled trial
SAS: Statistical Analysis System
SD: Standard Deviation
SF-36: Short Form-36
SOC: Stage of Change
TTM: Transtheoretical Model
VR: Virtual reality
