# Supplementary material for: The effect of active virtual reality gaming on physical activity behaviour and mental health in young men with mild to moderate depressive symptoms: a randomised controlled feasibility trial
Source: BMC Psychiatry. 2026 Feb 21;26:269. doi: 10.1186/s12888-026-07904-6 (PMC13032570; doi:10.1186/s12888-026-07904-6)
Supplement: Supplementary file 1 — Supplementary Material 1 [file 12888_2026_7904_MOESM1_ESM.docx]

#### Descriptive outcomes by group and time (Active vs Waitlist at Baseline and Endpoint).

| **Variable** | **Active — Baseline** | | | | **Active — Endpoint** | | | | **Waitlist — Baseline** | | | | **Waitlist — Endpoint** | | | |
| --- | --- | --- | --- | --- | --- | --- | --- | --- | --- | --- | --- | --- | --- | --- | --- | --- |
|  | **N** | **Mean (SD)** | **Median (IQR)** | **Min–Max** | **N** | **Mean (SD)** | **Median (IQR)** | **Min–Max** | **N** | **Mean (SD)** | **Median (IQR)** | **Min–Max** | **N** | **Mean (SD)** | **Median (IQR)** | **Min–Max** |
| PHQ-9 | 14 | 9.9 (2.6) | 9 (8–12) | 6–15 | 14 | 7 (5.1) | 6.5 (3–10) | 1–20 | 14 | 7.8 (3.8) | 8.5 (5–9) | 2–17 | 14 | 7.6 (4.8) | 6.5 (4–9) | 1–19 |
| DASS-21 Anxiety | 14 | 7.7 (4.8) | 7 (4–10) | 0–18 | 14 | 5.7 (5) | 5 (2–10) | 0–14 | 13 | 4.5 (4.6) | 4 (0–6) | 0–14 | 14 | 6.4 (5.6) | 6 (2–8) | 0–20 |
| DASS-21 Depression | 14 | 11.4 (5.5) | 9 (8–14) | 4–24 | 14 | 7.3 (6.5) | 7 (2–10) | 0–22 | 14 | 11.3 (7.2) | 10 (6–14) | 4–32 | 13 | 10.9 (7.4) | 10 (6–16) | 2–26 |
| DASS-21 Stress | 13 | 14.8 (9.6) | 12 (10–18) | 0–30 | 14 | 11.1 (9) | 10 (4–18) | 0–30 | 13 | 10.2 (8) | 8 (6–10) | 0–28 | 14 | 11.3 (7) | 9 (6–18) | 0–22 |
| PSQI | 14 | 8.1 (2.5) | 8 (7–9) | 4–14 | 14 | 8.3 (2.9) | 7.5 (6–9) | 5–16 | 14 | 6.9 (3) | 7.5 (5–9) | 1–11 | 14 | 6.9 (3.5) | 6 (5–10) | 2–14 |
| SF-36 Physical functioning | 14 | 87.9 (15.8) | 92.5 (80–100) | 45–100 | 14 | 92.1 (14.5) | 95 (94.4–100) | 45–100 | 14 | 95 (8.5) | 100 (95–100) | 70–100 | 14 | 95.4 (7.5) | 100 (95–100) | 75–100 |
| SF-36 Role function/physical | 14 | 82.1 (24.9) | 100 (75–100) | 25–100 | 14 | 87.5 (27.3) | 100 (75–100) | 0–100 | 14 | 76.8 (33.2) | 100 (50–100) | 0–100 | 14 | 82.1 (31.7) | 100 (75–100) | 0–100 |
| SF-36 Role function/emotional | 14 | 26.2 (26.7) | 33.3 (0–33.3) | 0–66.7 | 13 | 69.2 (39.6) | 100 (33.3–100) | 0–100 | 14 | 31 (33.2) | 33.3 (0–66.7) | 0–100 | 14 | 42.9 (40.1) | 33.3 (0–100) | 0–100 |
| SF-36 Energy/fatigue | 14 | 37.5 (13) | 37.5 (30–45) | 10–60 | 14 | 45.2 (15.2) | 50 (33.3–55) | 15–70 | 14 | 41.8 (21.1) | 40 (25–65) | 0–70 | 14 | 36.1 (22) | 37.5 (20–55) | 0–80 |
| SF-36 Emotional wellbeing | 14 | 60.3 (15.8) | 56 (48–76) | 36–84 | 14 | 68 (14.8) | 68 (64–80) | 32–88 | 14 | 61.4 (17.5) | 66 (44–72) | 28–84 | 14 | 62.6 (13.2) | 64 (56–72) | 40–80 |
| SF-36 Social functioning | 14 | 73.2 (23.4) | 75 (50–100) | 37.5–100 | 14 | 67.9 (28.5) | 81.3 (50–87.5) | 12.5–100 | 14 | 70.5 (20.6) | 75 (50–87.5) | 25–100 | 14 | 76.8 (20.1) | 81.3 (62.5–100) | 50–100 |
| SF-36 Pain | 14 | 83.4 (19.8) | 90 (77.5–100) | 35–100 | 14 | 78.9 (18.5) | 85 (70–90) | 35–100 | 14 | 87.7 (12.1) | 90 (77.5–100) | 67.5–100 | 14 | 82.1 (22.9) | 90 (77.5–100) | 32.5–100 |
| SF-36 General health | 14 | 56.8 (20.3) | 55 (50–75) | 30–90 | 14 | 62.1 (18.9) | 62.5 (50–75) | 25–100 | 14 | 60.7 (17.6) | 60 (50–70) | 35–100 | 14 | 63.6 (19.9) | 62.5 (50–80) | 30–95 |
| IPAQ | 14 | 1.4 (0.6) | 1 (1–2) | 1–3 | 14 | 2.1 (0.9) | 2 (1–3) | 1–3 | 14 | 1.3 (0.5) | 1 (1–2) | 1–2 | 14 | 1.8 (0.6) | 2 (1–2) | 1–3 |
| Stage of change | 14 | 2.6 (1.2) | 2 (2–3) | 1–5 | 14 | 3.1 (1.1) | 3 (2–4) | 1–5 | 14 | 2.4 (0.6) | 2 (2–3) | 1–3 | 14 | 2.5 (0.8) | 2.5 (2–3) | 1–4 |
| **EMI-2 subscales** | | | | | | | | | | | | | | | | |
| EMI-2 Stress management | 14 | 2.3 (1.2) | 2.1 (1.5–2.8) | 0–4.5 | 13 | 2.4 (1.5) | 2.5 (1.5–2.8) | 0–4.8 | 14 | 2 (1.5) | 2.3 (0.5–3.3) | 0–4.3 | 14 | 1.9 (1.1) | 1.8 (1–2.3) | 0.5–4 |
| EMI-2 Revitalisation | 14 | 2 (1.6) | 2.2 (0.7–3.3) | 0–4.3 | 13 | 2.4 (1.4) | 2.7 (1.7–3.3) | 0–4.3 | 14 | 2.1 (1.1) | 2 (1.3–3) | 0.3–4 | 14 | 2 (1.1) | 2.2 (1.3–3) | 0.3–3.7 |
| EMI-2 Enjoyment | 14 | 2 (1.5) | 2.1 (1–3) | 0–4.5 | 12 | 2.3 (1.4) | 2 (1.6–3.5) | 0–4.3 | 14 | 1.9 (1.5) | 1.8 (0.8–3.5) | 0–4.5 | 14 | 2.1 (1.6) | 2 (0.8–3.3) | 0–4.5 |
| EMI-2 Challenge | 14 | 2.5 (1.8) | 2.3 (1–4.3) | 0–5 | 13 | 2.4 (1.6) | 2.5 (1.5–3.5) | 0–5 | 14 | 1.8 (1.3) | 1.8 (0.8–3) | 0–4.3 | 13 | 2.2 (1.6) | 1.8 (1.5–3.3) | 0–4.8 |
| EMI-2 Social recognition | 13 | 1.1 (1.4) | 0.8 (0–1.8) | 0–4.3 | 13 | 1.5 (1.3) | 1.3 (1–2.3) | 0–4.3 | 14 | 1.6 (1.3) | 1.3 (1–2) | 0–5 | 13 | 1.2 (1.5) | 0.5 (0.3–1.5) | 0–4.3 |
| EMI-2 Affiliation | 13 | 1.2 (1.5) | 0.5 (0–1.8) | 0–3.8 | 13 | 1.4 (1.2) | 1.3 (0.5–1.8) | 0.3–4.3 | 14 | 1.7 (1.6) | 1.4 (0–3.3) | 0–4.3 | 14 | 1.9 (1.5) | 1.6 (0.5–3) | 0–4.8 |
| EMI-2 Competition | 13 | 1.7 (1.5) | 1.3 (0.3–3) | 0–4.5 | 13 | 1.4 (1.4) | 0.8 (0.5–2.3) | 0–4.8 | 14 | 1.6 (1.6) | 0.9 (0–3) | 0–4.3 | 14 | 1.6 (1.4) | 1.4 (0–2.8) | 0–4 |
| EMI-2 Health pressures | 14 | 0.6 (0.8) | 0 (0–1.3) | 0–2.3 | 13 | 0.8 (0.9) | 0.3 (0–1.7) | 0–2.3 | 14 | 0.8 (0.8) | 0.7 (0–1.7) | 0–2.3 | 13 | 0.7 (1.1) | 0 (0–0.7) | 0–3 |
| EMI-2 Ill-health avoidance | 14 | 2.8 (1.8) | 3.2 (1–4) | 0–5 | 13 | 3.4 (1.1) | 3.3 (3–4) | 1.3–5 | 14 | 2.8 (1.3) | 3 (2–3.7) | 0.7–5 | 14 | 2.7 (1.1) | 2.8 (2–3.3) | 1–5 |
| EMI-2 Positive health | 14 | 3.5 (1.5) | 4 (2.7–5) | 0.7–5 | 12 | 3.7 (1) | 3.7 (3.2–4.3) | 1.3–5 | 14 | 3.8 (0.9) | 3.7 (3–5) | 2.3–5 | 14 | 3.6 (0.9) | 3.3 (2.7–4.3) | 2.3–5 |
| EMI-2 Weight management | 14 | 3.6 (1.7) | 4.3 (3–4.8) | 0–5 | 13 | 3.6 (1.4) | 4 (3.3–4.8) | 0–5 | 14 | 2.7 (1.8) | 2.9 (1–4.5) | 0.5–5 | 14 | 2.7 (1.8) | 2.9 (1–4.8) | 0.3–5 |
| EMI-2 Appearance | 14 | 3.1 (1.3) | 3.3 (2.3–4.3) | 0–5 | 13 | 3.3 (1) | 3.8 (3–3.8) | 1–5 | 14 | 2.8 (1.1) | 3 (2–3.8) | 0.8–4 | 14 | 2.7 (1.2) | 3.3 (1.3–3.8) | 0.8–4 |
| EMI-2 Strength & endurance | 14 | 3.5 (1.8) | 4.1 (2.8–4.8) | 0–5 | 12 | 3.4 (1.6) | 3.8 (3.4–4.3) | 0–5 | 14 | 3.6 (1.1) | 4 (2.8–4.5) | 1.5–5 | 14 | 3.3 (1.2) | 3 (2.3–4.3) | 1.5–5 |
| EMI-2 Nimbleness | 14 | 2.8 (1.8) | 3.3 (1–4) | 0–5 | 13 | 3 (1.3) | 3 (2–4) | 0.3–5 | 14 | 2.7 (1) | 2.8 (2–3.7) | 1–4 | 14 | 2.6 (1.3) | 2.5 (1.3–4) | 0.7–5 |

Notes: N = number, SD = standard deviation, IQR = interquartile range. EMI-2 = Exercise Motivations Inventory–2; PHQ-9 = Patient Health Questionnaire–9; DASS-21 = Depression Anxiety Stress Scales–21; PSQI = Pittsburgh Sleep Quality Index; SF-36 = Short-Form Health Survey; IPAQ = International Physical Activity Questionnaire.
